# A model predictive control framework with fatigue life assessment for flywheel energy storage systems supported by active magnetic bearings

**DOI:** 10.1038/s41598-026-66926-y

**Published:** 2026-08-25

**Authors:** Amr Hashim, Mohamed L. Shaltout, Emad El-Kashif, Bassam A. Hussein

**Affiliations:** 1 Mechanical Design and Production Engineering Department, Faculty of Engineering, Cairo University, Giza, 12613 Egypt; 2 Mechatronics Department, Higher Technological Institute (HTI), 10th of Ramadan City, Egypt

**Keywords:** Renewable energy, Flywheel energy storage (FES), Active magnetic bearings (AMB), Adaptive model predictive control (AMPC), Fatigue loads, Energy science and technology, Engineering

## Abstract

Flywheel Energy Storage Systems (FESS) represent a promising technology in modern power systems, particularly for renewable energy applications that demand long service life, high power density, and fast dynamic response. To enhance operational efficiency, FESS are typically designed with a flywheel rotor supported by active magnetic bearings (AMBs), which are well suited for high-speed rotating applications. In this study, a model predictive control framework is proposed to regulate the dynamic behavior of a FESS equipped with AMBs. The proposed MPC framework simultaneously controls the AMBs and manages the charging and discharging processes of the FESS through a Permanent Magnet Synchronous Machine (PMSM) operating as a motor–generator and mechanically coupled to the flywheel rotor. Numerical simulation results demonstrate that the proposed control framework effectively regulates the energy exchange process while maintaining stable rotor suspension via the AMBs. Furthermore, the dynamic performance of the FESS and its control system is evaluated in terms of fatigue loads acting on the flywheel rotor induced by repeated charging and discharging cycles, which significantly influence the overall service life of the system.

## Introduction

In renewable energy applications, a durable and reliable energy storage system is essential; consequently, Flywheel Energy Storage Systems (FESS) have emerged as a particularly attractive solution. Compared to electrochemical batteries, supercapacitors, fuel cells, superconducting magnetic energy storage, and compressed air energy storage systems, FESS offer several advantages, including exceptionally long cycle life, high power density, relatively large energy storage capacity, and minimal routine maintenance requirements^1^. In a FESS, energy is stored mechanically in the form of kinetic energy of a rotating mass, where the storage capacity can be adjusted by modifying the flywheel’s rotational speed, geometry, or mass distribution^2,3^. To achieve frictionless operation and eliminate mechanical wear, FESS employs Active Magnetic Bearings (AMBs), which provide contact-free rotor support in contrast to conventional mechanical bearings^2,4–8^. Magnetic bearings support rotating components using electromagnetic forces without mechanical contact. However, the open-loop dynamics of AMB systems are inherently unstable, necessitating closed-loop feedback control to ensure stable operation. A typical AMB system consists of a rotor, electromagnetic actuators, power amplifiers, displacement sensors, and a digital controller^9,10^. Numerous control strategies have been proposed for AMB systems, including proportional–integral–derivative (PID) control, adaptive control, sliding mode control (SMC), model predictive control (MPC), fuzzy logic control (FLC), and robust control techniques. Recent research has increasingly focused on decentralized control architectures, optimal control formulations, and hybrid approaches that integrate MPC, SMC, and FLC to enhance disturbance rejection, improve stability margins, and maintain performance under modeling uncertainties^10,11^.

Model Predictive Control (MPC) is an advanced control strategy that employs a dynamic system model to predict future behavior and optimize control actions over a finite prediction horizon^12^. The performance of MPC strongly depends on accurate modeling and structural identification of the controlled plant^13^. For an AMB system along a single axis, the dynamics can be approximated by a second-order spring–mass–damper model. Based on this formulation, a prediction horizon is defined, and a state-space representation is developed using the state vector, control input, and system matrices derived from the AMB dynamics^11^. Flywheel energy storage systems are typically subjected to frequent charging and discharging cycles to meet long-term energy demands^14^. Consequently, the control system must exhibit high robustness and fast dynamic response, making MPC particularly well-suited for FESS applications^4^. Despite the advantages of frequent charging and discharging, speed variations during charging and discharging—especially under random load profiles—are critical contributors to fatigue damage. These variations induce fluctuating stresses that accumulate over time, thus reducing the service life of the flywheel rotor^15^. In FESS applications, MPC regulates control inputs by minimizing a predefined cost function while performing predictions over a limited horizon^16^. The primary control objectives include limiting rotor speed overshoots, preserving mechanical and thermal integrity, and suppressing excitation at critical frequencies. Furthermore, MPC enables the explicit incorporation of fatigue-related constraints, such as torque rate-of-change and acceleration limits, while accounting for actuator saturation and time-dependent system dynamics^12,17^.

The fatigue behavior of flywheel rotors has been extensively studied under various operating conditions. Multiaxial stress-based fatigue analyses have been conducted to evaluate rotor lifespan under different charging and discharging scenarios^18^. Energy-based fatigue approaches have shown that damage accumulation in steel flywheel rotors is highly sensitive to fluctuations in torque ramp rates and rotational speeds^18,19^. High centrifugal forces generated during flywheel operations become particularly severe under random speed variations. Using Miner’s rule combined with rainflow cycle counting, previous studies demonstrated that high-frequency stress reversals during frequent speed changes contribute significantly to cumulative fatigue damage, even when mean stress levels remain within allowable design limits^19^. Rainflow counting methods have also been applied to analyze impulsive loads on catcher bearings integrated with AMB systems during sudden rotor drops caused by excessive speed or control failure^20^. By applying rainflow counting to complex and random rotor loading histories, discrete stress cycles can be extracted and synchronized with fatigue damage models based on strain–life methods and material S–N (Wöhler) curves^21,22^. These techniques enable the evaluation of fluctuating-amplitude loading in flywheel systems subjected to random operational profiles, allowing the influence of control strategies to be quantified in analytical fatigue terms. Several studies have reported that fatigue life can be substantially extended by applying MPC-based control strategies that reduce both the frequency and severity of damaging stress cycles.

In^23^, a fatigue evaluation of an optimized steel flywheel rotor subjected to grid-scale energy demand was conducted using ANSYS finite-element simulations. The results showed that the rotor could operate for approximately 35,873 cycles (equivalent to about 98 years) at 6000 rpm; however, a significant reduction in lifespan was observed as rotational speed increased, with failure occurring at 10,000 rpm. This study emphasized the importance of structural optimization and precise speed control in extending flywheel lifespan, particularly for systems exposed to repeated charging and discharging cycles. Fatigue analysis techniques based on rainflow counting and Miner’s rule have also been applied to other rotational systems, such as Permanent Magnet Synchronous Motors (PMSMs), operating under variable loading conditions^24,25^. These methods typically involve peak-valley filtering, hysteresis processing, and stress-life modeling using piecewise linear approximations of Wöhler curves to predict fatigue behavior across low-, high-, and infinite-cycle regimes^9,22^. Implementations in MATLAB have further enabled control-integrated fatigue evaluation frameworks for rotating energy systems, including FESS^26^. In high-cycle rotating systems, advanced control strategies have been shown to significantly reduce fatigue loads. For example, state-space and predictive controllers applied to wind turbine systems using the Controls Advanced Research Technology (CART) platform demonstrated substantial reductions in tower bending and drivetrain torsional fatigue loads compared to conventional PID control. The effectiveness of predictive control in mitigating load fluctuations was confirmed through fatigue analyses based on Damage Equivalent Loads (DELs) and rainflow counting^27^. These findings support the integration of advanced control techniques to enhance fatigue endurance in FESS applications.

Previous investigations have addressed either the dynamic control of flywheel energy storage systems or the fatigue behavior of high-speed rotors. MPC-based FESS and AMB studies have mainly evaluated speed tracking, rotor displacement, disturbance rejection, control effort, and energy-management performance^4,10,17^. Separately, fatigue-oriented investigations have assessed flywheel or rotating-component life using prescribed speed or loading histories, finite-element stress analysis, S–N data, rainflow cycle counting, and cumulative-damage rules^18–20,23,26^. However, the cited studies do not systematically trace how the selection of MPC cost-function weights changes the torque and speed responses and, consequently, modifies the rotor stress-cycle distribution and cumulative fatigue damage within one integrated FESS–PMSM–AMB framework. This control-to-fatigue assessment gap motivates the present study. The present study develops an integrated control-to-fatigue evaluation framework for an active-magnetic-bearing-supported flywheel energy storage system. An MPC regulates the charge and discharge torque, flywheel speed, and power exchange through the PMSM, while a speed-dependent MPC formulation updates the AMB prediction model according to the measured rotor speed. The resulting controller-dependent speed history is converted into a nominal centrifugal-stress history, which is subsequently processed using rainflow cycle counting, mean-stress correction, and the Palmgren–Miner cumulative-damage rule. The contribution does not lie in introducing a new MPC formulation, rainflow algorithm, or fatigue-damage law. Rather, it lies in connecting these established methods so that the influence of controller tuning can be followed from the electrical and mechanical transient response to the resulting fatigue-damage indicator. Six combinations of speed-tracking and input-smoothing weights are evaluated to quantify the trade-off among tracking accuracy, torque smoothness, AMB loading, rotor dynamic response, cumulative fatigue damage, and fatigue-equivalent life. In the present work, fatigue damage is used as an offline criterion for comparing controller-tuning alternatives; it is not incorporated directly as an online MPC state or constraint.

The remainder of the paper is organized as follows. Section "System modeling" presents the AMB-supported rotor, PMSM, and FESS models. Section "Fatigue damage assessment" describes the nominal centrifugal-stress calculation, rainflow cycle counting, mean-stress correction, and cumulative fatigue-damage formulation. Section "Control methodology" presents the FESS MPC, the speed-dependent AMB controller, and the information flow among the control and fatigue models. Section "Simulation results" compares the dynamic and fatigue-related responses obtained using the six controller settings. Finally, Section "Conclusions" summarizes the main findings, limitations, and directions for further validation.

## System modeling

**Fig. 1:**
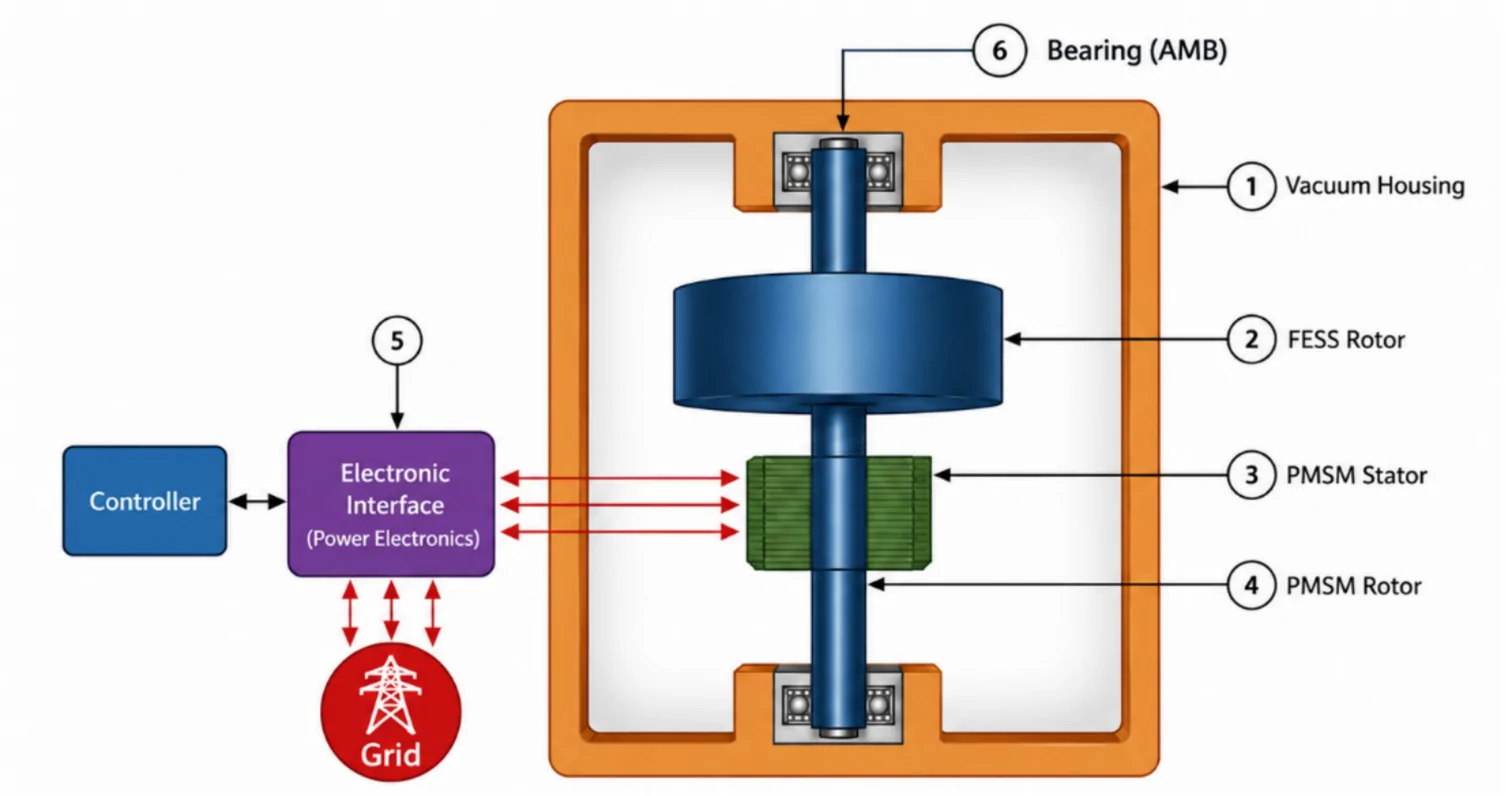
A schematic of FESS structure and components.

The dynamic system under investigation, as illustrated in Fig. 1, consists of a Flywheel Energy Storage System (FESS) with a rated energy capacity of 32 kWh and a nominal rotational speed of 9000 rpm, coupled with a Permanent Magnet Synchronous Machine (PMSM). The FESS model represents the operation of a high-speed rotating flywheel stabilized by Active Magnetic Bearings (AMBs), which provide contact-free support to minimize frictional losses and ensure smooth system operation. The rotor is enclosed within a rigid vacuum housing to enhance safety and maintain optimal operating conditions. The PMSM operates as a motor–generator, enabling bidirectional energy exchange between the flywheel rotor and the electrical grid. The amount of stored kinetic energy is directly dependent on the rotational speed of the flywheel.

The PMSM manages the electromagnetic interaction between the stator and rotor, with the power electronic interface enabling controlled bidirectional power exchange. The charging and discharging operations of the FESS are driven by grid demand, with the controller coordinating power management by regulating the flywheel’s rotational speed. During periods of low grid demand, the FESS absorbs excess energy by accelerating the flywheel rotor, whereas during periods of high demand, it supplies energy to the grid by decelerating the rotor. For the integrated system, the power delivered to the grid is managed as a function of the FESS discharge power and the grid’s real-time demand, thereby ensuring system stability and efficient utilization of the stored energy.

### Active magnetic bearing (AMB) model

**Fig. 2:**
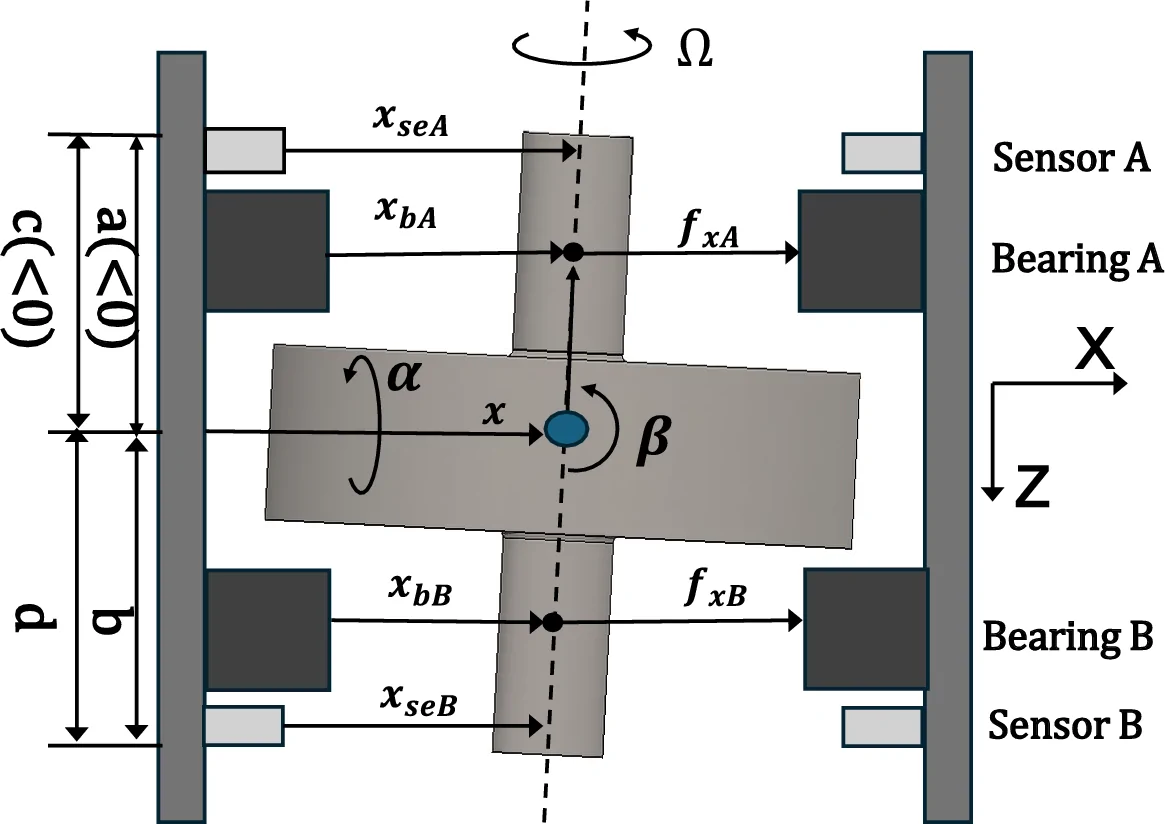
A schematic of the FESS rotor supported by two active magnetic bearing with displacement sensors.

The Flywheel Energy Storage System (FESS) under investigation consists of a high-speed rotor supported by two active radial magnetic bearings (AMBs) and enclosed within a vacuum chamber to minimize aerodynamic losses, as illustrated in Fig. 1. The rotor is driven by a Permanent Magnet Synchronous Machine (PMSM) that enables bidirectional energy transfer during charging and discharging operations. The AMBs generate electromagnetic forces to provide contact-free rotor suspension, thereby enabling mechanically frictionless operation. The system is modeled using a rigid rotor representation, in which the shaft and flywheel disk are treated as a lumped mass–inertia system with defined translational and rotational degrees of freedom. The rotor dynamics are derived using the Newton–Euler equations of motion, incorporating the effects of magnetic bearing forces, gyroscopic moments, and mass unbalance. As shown in Fig. 2**,** a right-handed inertial coordinate system *OXYZ* is fixed to the stationary vacuum housing, with the *Z*-axis coincident with the nominal rotor spin axis. The variables *x* and *y*, measured in meters denote the lateral translations of the rotor center of mass along the *X*- and *Y*-axes, respectively. The axial displacement of the shaft in *Z*-axis is neglected. The variables *α* and *β*, measured in radians, denote small rotor tilting angles about the *X*- and *Y*-axes. Positive rotations follow the right-hand rule. Bearing locations *A* and *B* are measured along the rotor axis relative to the rotor center of mass. Bold lowercase symbols denote vectors, bold uppercase symbols denote matrices, and italic symbols denote scalar quantities. Unless otherwise stated, all quantities are expressed in SI units.

For control design and transient response analysis, the rotor–bearing dynamics are formulated in state-space form. A convenient approach to obtain the linearized equations of motion for the configuration shown in Fig. 2 is to select the small center-of-mass displacements *x* and *y*, along with the Euler angles *α* and *β*, as generalized coordinates, assembled in the state vector $\mathrm{q}$. For the rigid-rotor approximation shown in Fig. 2, the generalized-coordinate vector leading to the system relations reported in^28^ is defined as,

$$\mathrm{q}={\left[\begin{array}{cccc}\beta & x& -\alpha & y\end{array}\right]}^{T}\in {\mathbb{R}}^{4},$$

where *x* and *y* are lateral center-of-mass displacements in meters and *α* and *β* are small angular displacements in radians. The negative sign associated with *α* follows the selected right-handed coordinate convention and preserves consistency between the *x*- and *y*-plane equations. The generalized-force vector is defined as follows,

$${\mathrm{u}}_{f}={\left[\begin{array}{cccc}{f}_{xA}& {f}_{xB}& {f}_{yA}& {f}_{yB}\end{array}\right]}^{T}\in {\mathbb{R}}^{4},$$

where each component is an AMB force measured in Newtons. The measured output vector is,

$${\mathbf{y}}_{AMB}={\left[\begin{array}{cccc}{x}_{seA}& {x}_{seB}& {y}_{seA}& {y}_{seB}\end{array}\right]}^{T}\in {\mathbb{R}}^{4},$$

where the four sensor outputs are measured lateral displacements in meters. The state-space representation of the rotor-bearing dynamics can be described as follows,

1-a $$\mathrm{M}\ddot{\mathrm{q}}+\mathrm{G}(\Omega )\dot{\mathrm{q}}={\mathrm{B}}_{f}{\mathrm{u}}_{f}$$

1-b $${\mathrm{y}}_{AMB}={\mathrm{C}}_{s}\mathrm{q}$$

where $\mathbf{M}\in {\mathbb{R}}^{4\times 4}$ is the generalized mass–inertia matrix and $\mathbf{G}(\Omega )\in {\mathbb{R}}^{4\times 4}$ is the speed-dependent gyroscopic matrix. The first and third rows of these matrices correspond to rotational equations, whereas the second and fourth rows correspond to translational equations. Consequently, the matrix entries have generalized units consistent with the mixed angular and translational coordinates. The matrix ${\mathbf{B}}_{f}\in {\mathbb{R}}^{4\times 4}$ maps the four bearing forces to the generalized moments and forces acting at the rotor center of mass. Its coefficients *a* and *b*, measured in meters, are the signed axial distances from the center of mass to bearings *A* and *B*, as shown in Fig. 2. The sensor matrix ${\mathbf{C}}_{s}\in {\mathbb{R}}^{4\times 4}$ maps the generalized coordinates to the four measured lateral displacements. Its coefficients *c* and *d*, measured in meters are the signed axial distances from the rotor center of mass to the displacement sensors. The vectors and matrices presented in Eq. (1) are detailed as follows:

$$\mathrm{M}=\left[\begin{array}{cccc}{I}_{y}& 0& 0& 0\\ 0& m& 0& 0\\ 0& 0& {I}_{x}& 0\\ 0& 0& 0& m\end{array}\right], \mathrm{G}=\left[\begin{array}{cccc}0& 0& {I}_{z}\Omega & 0\\ 0& 0& 0& 0\\ -{I}_{z}\Omega & 0& 0& 0\\ 0& 0& 0& 0\end{array}\right],$$

$${\mathrm{B}}_{f}=\left[\begin{array}{cccc}a& b& 0& 0\\ 1& 1& 0& 0\\ 0& 0& a& b\\ 0& 0& 1& 1\end{array}\right], {\mathrm{C}}_{s}=\left[\begin{array}{cccc}c& 1& 0& 0\\ d& 1& 0& 0\\ 0& 0& c& 1\\ 0& 0& d& 1\end{array}\right],$$

where the rotor mass is denoted as *m*, and ${I}_{x, } {I}_{y}$, and ${I}_{z}$ are the rotor mass moments of inertia about the *X*-axis, *Y*-axis, and *Z*-axis, respectively. The rotor speed is denoted as *Ω* in rad/s, ${f}_{yA}$ and ${f}_{yB}$ are magnetic forces generated by bearings *A* and *B* in the *Y*-direction, ${f}_{xA}$ and ${f}_{xB}$ are magnetic forces generated by bearings *A* and *B* in the *X*-direction, and *a* and *b* are geometric coefficients representing the axial distances between the rotor center of mass and bearings *A* and *B*, respectively. While *c* and *d* are sensor location coefficients, that map rotor translations and tilting angles to sensor positions.

Under a small-perturbation model, the magnetic-bearing force ${\mathbf{u}}_{f}$ is approximated as a linear function of the rotor displacements at the bearing and the coil currents, parameterized by the force–current coefficient ${k}_{i}$ and the force–displacement coefficient ${k}_{s}$. These coefficients may differ among bearings, but symmetry implies equal values in the *x* and *y* directions of a given bearing. Accordingly, the input force vector can be written as:

2-a $${\mathrm{u}}_{f}=-{\mathrm{K}}_{s}{\mathrm{q}}_{b}+{\mathrm{K}}_{i}\mathrm{i},$$

2-b $${\mathrm{u}}_{f}=\left[\begin{array}{c}{f}_{xA}\\ {f}_{xB}\\ {f}_{yA}\\ {f}_{yB}\end{array}\right]=-\left[\begin{array}{cccc}{k}_{sA}& 0& 0& 0\\ 0& {k}_{sB}& 0& 0\\ 0& 0& {k}_{sA}& 0\\ 0& 0& 0& {k}_{sB}\end{array}\right]\left[\begin{array}{c}{x}_{bA}\\ {x}_{bB}\\ {y}_{bA}\\ {y}_{bB}\end{array}\right]+\left[\begin{array}{cccc}{k}_{iA}& 0& 0& 0\\ 0& {k}_{iB}& 0& 0\\ 0& 0& {k}_{iA}& 0\\ 0& 0& 0& {k}_{iB}\end{array}\right]\left[\begin{array}{c}{i}_{xA}\\ {i}_{xB}\\ {i}_{yA}\\ {i}_{yB}\end{array}\right],$$

where ${\mathbf{q}}_{b}\in {\mathbb{R}}^{4}$ contains the lateral rotor displacements at bearings *A* and *B*, measured in meters, and $\mathbf{i}\in {\mathbb{R}}^{4}$ contains the four control currents, measured in amperes. The matrices ${\mathbf{K}}_{s}\in {\mathbb{R}}^{4\times 4}$ and ${\mathbf{K}}_{i}\in {\mathbb{R}}^{4\times 4}$ are diagonal matrices containing the force–displacement coefficients ${k}_{s}$, in $\mathrm{N}/\mathrm{m}$, and force–current coefficients ${k}_{i}$, in $\mathrm{N}/\mathrm{A}$, respectively. Thus, both terms on the right-hand side of Eq. (2) have units of force in Newtons. The closed-loop dynamics in a state space representation can be described as follows,

3-a $$\mathrm{M}\ddot{\mathrm{q}}+\mathrm{G}\left(\Omega \right)\dot{\mathrm{q}}={\mathrm{B}}_{f}\left(-{\mathrm{K}}_{\mathrm{s}}{\mathrm{q}}_{\mathrm{b}}+{\mathrm{K}}_{\mathrm{i}}\mathrm{i}\right),$$

3-b $${\mathrm{y}}_{AMB}={\mathrm{C}}_{s}\mathrm{q}.$$

By transforming the bearing coordinates ${\mathrm{q}}_{b}$ to the center-of-mass coordinates $\mathbf{q}$ using the linear transformation ${\mathrm{T}}_{bs}$, whose entries depend on the geometric parameters *a* and *b* described in Fig. 2, gives,

4 $${\mathrm{q}}_{b}=\left[\begin{array}{c}{x}_{bA}\\ {x}_{bB}\\ {y}_{bA}\\ {y}_{bB}\end{array}\right]=\left[\begin{array}{cccc}a& 1& 0& 0\\ b& 1& 0& 0\\ 0& 0& a& 1\\ 0& 0& b& 1\end{array}\right]\left[\begin{array}{c}\beta \\ x\\ -\alpha \\ y\end{array}\right].$$

The bearing-coordinate vector ${\mathrm{q}}_{b}$ is related to the center-of-mass coordinate vector $\mathrm{q}$ through the following relation,

$${\mathrm{q}}_{b}={\mathrm{T}}_{bs}\mathrm{q},{ \mathrm{T}}_{bs}\in {\mathbb{R}}^{4\times 4}.$$

For the selected rigid-rotor geometry, ${\mathrm{T}}_{bs}={\mathrm{B}}_{f}^{T}$. This relation follows from geometric compatibility and work reciprocity: the same axial distances that map bearing forces to generalized forces also map center-of-mass translations and rotations to rotor displacements. Substituting Eq. (4) into Eq. (3) yields a differential matrix equation expressed exclusively in the CG coordinates $\mathrm{q}$ as follows,

5 $$\mathrm{M}\ddot{\mathrm{q}}+\mathrm{G}\left(\Omega \right)\dot{\mathrm{q}}=-{\mathrm{B}}_{f}{\mathrm{K}}_{\mathrm{s}}{\mathrm{B}}_{f}^{T}\mathrm{q}+{{\mathrm{B}}_{f}\mathrm{K}}_{\mathrm{i}}\mathrm{i}=-{\mathrm{K}}_{\mathrm{s}\mathrm{s}}\mathrm{q}+{\mathrm{B}}_{f}{\mathrm{K}}_{\mathrm{i}}\mathrm{i},$$

where the negative bearing-stiffness matrix ${\mathrm{K}}_{ss}$ is expressed in CG coordinates. As with any displacement proportional term, this negative stiffness contribution can be shifted to the left-hand side of Eq. (5), leaving only the control-current term on the right-hand side, giving:

6 $$\mathrm{M}\ddot{\mathrm{q}}+\mathrm{G}(\Omega )\dot{\mathrm{q}}+{\mathrm{K}}_{\mathrm{s}\mathrm{s}}\mathrm{q}={{\mathrm{B}}_{f}\mathrm{K}}_{\mathrm{i}}\mathrm{i}$$

Since ${\mathbf{K}}_{s}$ is a diagonal matrix, Eq. (5) shows that the transformed negative bearing-stiffness matrix ${\mathbf{K}}_{ss}$ is symmetric,

7-a $${\mathrm{K}}_{\mathrm{s}\mathrm{s}}^{T}={({\mathrm{B}}_{f}{\mathrm{K}}_{\mathrm{s}}{\mathrm{B}}_{f}^{T})}^{T}={\mathrm{BK}}_{\mathrm{s}}^{T}{\mathrm{B}}_{f}^{T}={\mathrm{K}}_{\mathrm{s}\mathrm{s}}$$

7-b $${\mathbf{K}}_{\mathbf{s}\mathbf{s}}=\left[\begin{array}{cccc}{k}_{sA}{a}^{2}+{k}_{sB}{b}^{2}& {k}_{sA}a+{k}_{sB}b& 0& 0\\ {k}_{sA}a+{k}_{sB}b& {k}_{sA}+{k}_{sB}& 0& 0\\ 0& 0& {k}_{sA}{a}^{2}+{k}_{sB}{b}^{2}& {k}_{sA}a+{k}_{sB}b\\ 0& 0& {k}_{sA}a+{k}_{sB}b& {k}_{sA}+{k}_{sB}\end{array}\right]$$

The effect of an added unbalanced mass on the rotor can be represented as follows,

8-a $${M}_{\varepsilon y}={m}_{u}r{\Omega }^{2}l\mathrm{cos}\Omega t,$$

8-b $${F}_{ex}={m}_{u}r{\Omega }^{2}\mathit{cos}\Omega t ,$$

8-c $${M}_{\varepsilon x}={m}_{u}r{\Omega }^{2}l\mathrm{sin}\Omega t,$$

8-d $${F}_{ey}={m}_{u}r{\Omega }^{2}\mathit{sin}\Omega t,$$

where ${M}_{\varepsilon y}$ and ${M}_{\varepsilon x}$ denote the moments about the—*β*and *α*-axes, respectively, while ${F}_{ey}$ and ${F}_{ex}$ are the excitation forces in the—*Y*and *X*—directions. The unbalanced mass is denoted as ${m}_{u}$, *r* is the rotor radius, and *l* is the eccentricity. The resulting system dynamics under this imbalance are given as follows,

9-a $$\mathrm{M}\ddot{\mathrm{q}}+\mathrm{G}\left(\Omega \right)\dot{\mathrm{q}}+{\mathrm{K}}_{\mathrm{s}\mathrm{s}}\mathrm{q}={{\mathrm{B}}_{f}\mathrm{K}}_{\mathrm{i}}\mathrm{i}+ {\mathrm{F}}_{unb},$$

9-b $${\mathrm{F}}_{unb}=\left[\begin{array}{c}\begin{array}{c}{M}_{\varepsilon y}\\ {F}_{ex}\\ {M}_{\varepsilon x}\end{array}\\ {F}_{ey}\end{array}\right]=\left[\begin{array}{c}{m}_{u}r{\Omega }^{2}l\mathrm{cos}\Omega t\\ {m}_{u}r{\Omega }^{2}\mathit{cos}\Omega t\\ {m}_{u}r{\Omega }^{2}l\mathrm{sin}\Omega t\\ {m}_{u}r{\Omega }^{2}\mathit{sin}\Omega t\end{array}\right].$$

To obtain a first-order state-space model, the AMB rotor state vector is defined as,

$${\mathrm{x}}_{AMB}={\left[\begin{array}{cc}{\mathrm{q}}^{T}& {\dot{\mathrm{q}}}^{T}\end{array}\right]}^{T}\in {\mathbb{R}}^{8}.$$

The state components have the units [rad, m, rad, m, rad/s, m/s, rad/s, m/s]. Equation (9) can then be expressed as,

10 $${\dot{\mathrm{x}}}_{AMB}={\mathrm{A}}_{AMB}(\Omega ){\mathrm{x}}_{AMB}+{\mathrm{B}}_{AMB}\mathrm{i}+{\mathrm{E}}_{AMB}{\mathrm{F}}_{unb},$$

11 $${\mathrm{y}}_{AMB}={\mathrm{C}}_{AMB}{\mathrm{x}}_{AMB}+{\mathrm{D}}_{AMB}\mathrm{i},$$

where

$${\mathrm{A}}_{AMB}(\Omega )=\left[\begin{array}{cc}{0}_{4\times 4}& {\mathrm{I}}_{4\times 4}\\ -{\mathrm{M}}^{-1}{\mathrm{K}}_{ss}& -{\mathrm{M}}^{-1}\mathrm{G}(\Omega )\end{array}\right]\in {\mathbb{R}}^{8\times 8}, {\mathrm{B}}_{AMB}=\left[\begin{array}{c}{0}_{4\times 4}\\ {\mathrm{M}}^{-1}{\mathrm{B}}_{f}{\mathrm{K}}_{i}\end{array}\right]\in {\mathbb{R}}^{8\times 4},$$

$${\mathrm{E}}_{AMB}=\left[\begin{array}{c}{0}_{4\times 4}\\ {\mathrm{M}}^{-1}\end{array}\right]\in {\mathbb{R}}^{8\times 4} , {\mathrm{C}}_{AMB}=\left[\begin{array}{cc}{\mathrm{C}}_{s}& {0}_{4\times 4}\end{array}\right]\in {\mathbb{R}}^{4\times 8}, {\mathrm{D}}_{AMB}={0}_{4\times 4}$$

The disturbance vector is expressed as,

$${\mathrm{F}}_{unb}={\left[\begin{array}{cccc}{M}_{\varepsilon y}& {F}_{ex}& {M}_{\varepsilon x}& {F}_{ey}\end{array}\right]}^{T},$$

And it comprises generalized moments and forces with units ${\left[\mathrm{N}.\mathrm{m},\hspace{0.17em}\mathrm{N},\hspace{0.17em}\mathrm{N}.\mathrm{m},\hspace{0.17em}\mathrm{N}\right]}^{T}$. The dependence of ${\mathbf{A}}_{AMB}$ on the measured speed *Ω* arises from the gyroscopic matrix $\mathbf{G}(\Omega )$. For numerical simulation, the adopted system model parameters are shown in Table 1.

**Table 1:** The adopted rotor-bearing system model parameters.

Parameter	Value	Unit
FESS mass,*m*	2353.45	kg
Moments of inertia, *I*_*x*_ = *I*_*y*_	192.5	kg.m^2^
Moments of inertia, *I*_*z*_	311.7	kg.m^2^
*b* = *− a*	0.45	m
*d* = *− c*	0.5	m
Force–displacement constant,* k*_*sA*_ = *k*_*sB*_	1 × 10^7^	N/m
Force-current constant,* k*_*i*A_ = *k*_*iB*_	250	N/A
FESS radius	0.535	m
unbalanced mass, ${m}_{u}$	2.223 × 10^–3^	kg
Eccentricity, *l*	0.13825	m
Simulation time	4.0 × 10^5^	s

### Permanent magnet synchronous machine (PMSM) model

Permanent-magnet synchronous machines are typically modeled and controlled using field-oriented control^29^ in the *d-q* frame. The Clarke-transform maps the three-phase stator voltages $({V}_{a},{V}_{b},{V}_{c})$ to the stationary $\alpha -\beta$ frame, and the Park-transform rotates them into the rotor-aligned $({V}_{d},{V}_{q})$ coordinates required by the two-axis PMSM model. An appropriate reference-frame choice removes time-varying coupling in the phase equations and markedly simplifies analysis and controller synthesis. The adopted PMSM specifications are shown in Table 2 as in accordance with the PMSM specifications in^14,30^. The PMSM equations are expressed in the rotor-synchronous *d-q* reference frame. The *d*-axis is aligned with the permanent-magnet flux vector, and the *q*-axis is displaced by ${90}^{\circ }$ electrical from the *d*-axis. The stator voltages ${v}_{d}$ and ${v}_{q}$ are measured in volts, the stator currents ${i}_{d}$ and ${i}_{q}$ in amperes, the stator resistance ${R}_{s}$ in ohms, the inductances ${L}_{d}$ and ${L}_{q}$ in Henries, and the flux linkages ${\lambda}_{d}$, ${\lambda}_{q}$, and ${\lambda}_{f}$ in Weber’s. The electrical angular speed ${\omega}_{r}$ and mechanical flywheel speed *Ω* are expressed in $\mathrm{r}\mathrm{a}\mathrm{d}/\mathrm{s}$.

**Table 2:** The adopted PMSM specifications.

Parameter	Value	Unit
Rated power	128	kW
Rated torque	136	N.m
Maximum speed	9000	rpm
DC-link voltage	380	V

The mathematical model PMSM can be presented as described in^14,29,31,32^. The park transformation and its inverse, which convert three-phase voltage into (*d-q*) voltage, are described as follows,

12 $$\left[\begin{array}{c}{V}_{d}\\ {V}_{q}\\ {V}_{0}\end{array}\right]=\frac{2}{3}\left[\begin{array}{ccc}cos\theta & cos\left(\theta -\frac{2\pi }{3}\right)& cos\left(\theta +\frac{2\pi }{3}\right)\\ -sin\theta & -sin\left(\theta -\frac{2\pi }{3}\right)& -sin\left(\theta +\frac{2\pi }{3}\right)\\ \frac{1}{2}& \frac{1}{2}& \frac{1}{2}\end{array}\right]\left[\begin{array}{c}{V}_{a}\\ {V}_{b}\\ {V}_{c}\end{array}\right],$$

13 $$\left[\begin{array}{c}{V}_{a}\\ {V}_{b}\\ {V}_{c}\end{array}\right]=\left[\begin{array}{ccc}cos\theta & -sin\theta & 1\\ cos\left(\theta -\frac{2\pi }{3}\right)& -sin\left(\theta -\frac{2\pi }{3}\right)& 1\\ cos\left(\theta +\frac{2\pi }{3}\right)& -sin\left(\theta +\frac{2\pi }{3}\right)& 1\end{array}\right]\left[\begin{array}{c}{V}_{d}\\ {V}_{q}\\ {V}_{0}\end{array}\right],$$

where *V*₀ (zero-voltage component) is generally omitted, and θ represents the phase (*a*) electrical angle with regard to the reference frame. Effective PMSM control requires a tractable mathematical model. The full three-phase, time-varying formulation is rarely used in controller synthesis because its periodic coefficients complicate analysis. Instead, the machine is represented in a two-axis frame, aligned with the direct and quadrature axes, which yields a time-invariant model suitable for control design. A standard PMSM model comprises stator flux relations, stator voltage equations, mechanical dynamics, and electromagnetic torque. In the rotor-synchronous *d-q* frame, the stator voltage equations take the form,

14 $${v}_{q}={R}_{s}{i}_{q}+{\omega}_{r}{\lambda}_{d}+\frac{d}{dt}{\lambda}_{q},$$

15 $${v}_{d}={R}_{s}{i}_{d}-{\omega}_{r}{\lambda}_{q}+\frac{d}{dt}{\lambda}_{d}.$$

The flux linkage formulas are expressed as follows,

16 $${\lambda}_{q}={L}_{q}{i}_{q},$$

17 $${\lambda}_{d}={L}_{d}{i}_{d}+{\lambda}_{f}.$$

In Eqs. (14) to (17), ${R}_{s}$ is the stator phase resistance measured in ohms; ${L}_{d}$ and ${L}_{q}$ are the direct- and quadrature-axis inductances measured in Henry; ${i}_{d}$ and ${i}_{q}$ are the corresponding stator-current components measured in amperes; ${v}_{d}$ and ${v}_{q}$ are the stator-voltage components measure in volts; ${\lambda}_{d}$, ${\lambda}_{q}$, and ${\lambda}_{f}$ are the stator and permanent-magnet flux linkages measured in Weber; and ${\omega}_{r}$ is the electrical angular speed measure in rad/s. By substituting Eqs. (16) and (17) into Eqs. (14) and (15), the following relations can be obtained,

18 $${v}_{q}={R}_{s}{i}_{q}+{\omega}_{r}\left({L}_{d}{i}_{d}+{\lambda}_{f}\right)+\frac{d}{dt}\left({L}_{q}{i}_{q}\right),$$

19 $${v}_{d}={R}_{s}{i}_{d}-{\omega}_{r}{L}_{q}{i}_{q}+\frac{d}{dt}\left({L}_{d}{i}_{d}+{\lambda}_{f}\right).$$

In the *d*–*q* reference frame, the PMSM electromagnetic torque is given by:

20 $${T}_{e}=\frac{3}{4}P\left({\lambda}_{d}{i}_{q}-{\lambda}_{q}{i}_{d}\right).$$

In Eq. (20), ${T}_{e}$ is the electromagnetic torque in $\mathrm{N}.\mathrm{m}$, and *P* is the total number of machine poles. Therefore, *P/2* is the number of pole pairs. The coefficient *3P/4* is equivalent to the conventional coefficient *3p/2*, where *p=P/2*.

The mechanical torque balance is affected by the mechanical rotational speed by the following equation:

21 $$J\dot{\Omega }={T}_{e}-{T}_{L}-\gamma\Omega ,$$

where *J* is the total rotating inertia referred to the machine shaft measured in $\mathrm{k}\mathrm{g}\mathrm{.}{\mathrm{m}}^{2}$, ${T}_{L}$ is the applied mechanical load torque measured in $\mathrm{N}.\mathrm{m}$, and *γ* is the viscous-friction coefficient measured in $\mathrm{N}.\mathrm{m}\mathrm{.}\mathrm{s}/\mathrm{r}\mathrm{a}\mathrm{d}$. The mechanical speed is related to the electrical speed by:

22 $$\Omega =\frac{2}{P}{\omega}_{r},$$

which connects the electrical speed used in the PMSM equations to the mechanical speed used in the FESS and AMB models.

### Flywheel energy storage system (FESS) model

A flywheel energy storage system (FESS) stores and releases its kinetic energy by connecting the flywheel rotor to a fast PMSM^14^, allowing it to switch energy between electrical and mechanical forms in both directions. It is well suited to short-duration storage. The FESS operates in three modes: charging, stand-by, and discharging^33^. In charging mode, the machine functions as a motor, draws electrical power, accelerates the rotor to a prescribed speed, and stores energy as kinetic energy. In stand-by mode, the rotor spins at a set speed with no external load. In discharging mode, the rotor decelerates and drives the machine as a generator; the controller adjusts the released kinetic energy into electrical output^34^. In this study, the variable-speed electric machine and its power electronics are represented by a first-order model with dynamics that are fast relative to the mechanical plant^34^. The FESS mechanics are then approximated by a single-inertia rotational model as follows:

23 $$\dot{\Omega }=\frac{1}{{J}_{f}}\left[{T}_{f}-{T}_{\mathrm{l}\mathrm{o}\mathrm{s}\mathrm{s}}(\Omega )\right],$$

where ${T}_{\mathrm{l}\mathrm{o}\mathrm{s}\mathrm{s}}={\eta}_{f}\mid {T}_{f}\mid$ represents the equivalent mechanical-loss torque and ${\eta}_{f}$ is dimensionless. In Eq. (23), ${J}_{f}$ is the combined rotational inertia of the flywheel and machine rotor measured in $\mathrm{k}\mathrm{g}.{\mathrm{m}}^{2}$, and ${T}_{f}$ is the controlled flywheel torque measured in $\mathrm{N}\mathrm{.}\mathrm{m}$. Positive ${T}_{f}$ denotes charging and rotor acceleration, whereas negative ${T}_{f}$ denotes discharging and rotor deceleration. The stored kinetic energy and mechanical flywheel power can be calculated as follows,

$${E}_{f}=\frac{1}{2}{J}_{f}{\Omega }^{2},$$

$${P}_{f}={T}_{f}\Omega ,$$

where ${E}_{f}$ is measured in joules and ${P}_{f}$ in watts. These relations provide the direct interface between the PMSM torque output and the charging or discharging state of the FESS.

## Fatigue damage assessment

Renewable energy sources, such as solar and wind power, often produce output fluctuations that do not match with grid demand in energy-intensive systems. Flywheel energy storage systems (FESS) are employed to reduce this mismatch by smoothing power to reduce the gap between generation and consumption. Among available storage technologies, flywheels are a suitable selection and attractive due to their environmentally benign characteristics^23^. However, the repeated charging and discharging required by power management strategies subject the rotor to stochastic mechanical loading. The present fatigue calculation uses the nominal centrifugal stresses of an idealized, homogeneous, isotropic, constant-thickness solid disk^35^, as follows,

24 $${\sigma}_{r}=\frac{3+v}{8}\rho {\Omega }^{2}\left({{r}_{0}}^{2}-{r}^{2}\right),$$

25 $${\sigma}_{\theta }=\frac{3+{{v}}}{8}\rho {\Omega }^{2}({{r}_{0}}^{2}-\frac{1+3v}{3+{{v}}}{r}^{2}),$$

where ${\sigma}_{r}$ is the radial stress and ${\sigma}_{\theta }$ is the hoop stress. The variable *r* denotes the radial coordinate measured from the disk center, whereas ${r}_{0}$ denotes the disk outer radius. The rotor angular speed *Ω* is expressed in $\mathrm{r}\mathrm{a}\mathrm{d}/\mathrm{s}$, the density *ρ* in $\mathrm{k}\mathrm{g}/{\mathrm{m}}^{3}$, and Poisson’s ratio *ν* is dimensionless. At the center of the disk, where *r=0*, the stresses are equal and maximum, given by:

26 $${\left[{\sigma}_{{{r}}}\right]}_{{{r}}=0}={\left[{\sigma}_{\theta }\right]}_{{{r}}=0}= {\left[\sigma \right]}_{{{m}}{{a}}{{x}}}=\frac{3+{{v}}}{8}\rho {\Omega }^{2}{{r}_{0}}^{2}$$

The time-varying stress input used in the fatigue algorithm is therefore,

$${\sigma}_{\mathrm{m}\mathrm{a}\mathrm{x}}\left(t\right)=\frac{3+\nu }{8}\rho {r}_{0}^{2}{\Omega }^{2}\left(t\right).$$

In the numerical fatigue calculation, ${\sigma}_{\mathrm{m}\mathrm{a}\mathrm{x}}(t)$ is converted from pascals to megapascals before it is evaluated against the material S–N data.

The stress formulation adopted in Eqs. (24) to (26) represents an idealized, homogeneous, isotropic, constant-thickness solid disk subjected predominantly to centrifugal loading, with the maximum nominal radial and circumferential stresses evaluated at the disk center. This analytical model was selected deliberately because the objective of the present study is to provide a comparative, control-oriented evaluation of the fatigue consequences associated with different MPC/AMPC tuning choices under identical rotor geometry, material properties, operating profiles, and fatigue parameters. Accordingly, the calculated cumulative damage and fatigue-equivalent life are intended to indicate relative differences among the investigated control settings rather than to constitute absolute service-life predictions for a specific manufactured flywheel. The present model does not explicitly account for local stress concentrations associated with the shaft–hub interface, bore, keyway, fillets, or other geometric discontinuities, nor does it include manufacturing-induced residual stresses, surface condition, dimensional tolerances, material-property variability, thermal effects, or multiaxial stress states away from the disk center. These factors may significantly affect the local fatigue response and should therefore be incorporated when translating the comparative trends reported here into design-level lifetime estimates. A geometry-resolved finite-element analysis, combined with an appropriate multiaxial fatigue criterion and experimentally characterized material and manufacturing parameters, is consequently identified as an important extension of the present work.

Fatigue damage accumulates over the service life of the FESS because of fluctuating mechanical loading. These load variations can be decomposed into individual hysteresis cycles by pairing local minima and maxima in the stress or strain time series with rainflow counting. Each cycle is then characterized by its mean level and amplitude. Under the Palmgren–Miner linear damage rule, the contribution of each cycle is assumed to add linearly, so that the total accumulated damage (*D*) is obtained by summing the damage of all counted cycles^15,36^ as follows,

27 $$D=\sum_{i}\frac{{n}_{i}}{{N}_{i}\left({L}_{i}^{RF}\right)},$$

where ${N}_{i}\left(\cdot \right)$ denotes number of cycles to failure, ${n}_{i}$ is the counted number of cycles, and ${L}_{i}^{RF}$ represents the load range of the *i*^th^ rainflow cycle about a fixed mean load. The dependence of the allowable cycles to failure on the load range, i.e. the S–N (Wöhler curve) relationship, is described by the following model,

28 $${N}_{i}={\left(\frac{{L}^{ult}-|{L}^{MF}|}{\frac{1}{2}{L}_{i}^{RF}}\right)}^{m} ,$$

29 $${L}_{i}^{RF}={L}_{i}^{R}\left(\frac{{L}^{ult}-|{L}^{MF}|}{{L}^{ult}-|{L}_{i}^{M}|}\right),$$

where ${L}^{ult}$ represents the ultimate design load of the component, ${L}^{MF}$ denotes the fixed mean load, ${L}_{i}^{R}$ is the ${i}^{th}$ cycle’s range about a load mean of ${L}_{i}^{M}$, and *m* is the Whöler exponent, which is a material-specific parameter associated with the fatigue characteristics of the component. Equation (29) assumes that fatigue cycles develop around a constant mean load. In practice, however, the load cycles occur over a range of mean load levels. To account for this variability, the load ranges of the fatigue cycles are adjusted so that each cycle can be evaluated as if it occurred about a fixed mean load. This adjustment follows the Goodman correction, applied here with a Goodman exponent that equals to unity^36^.

**Fig. 3:**
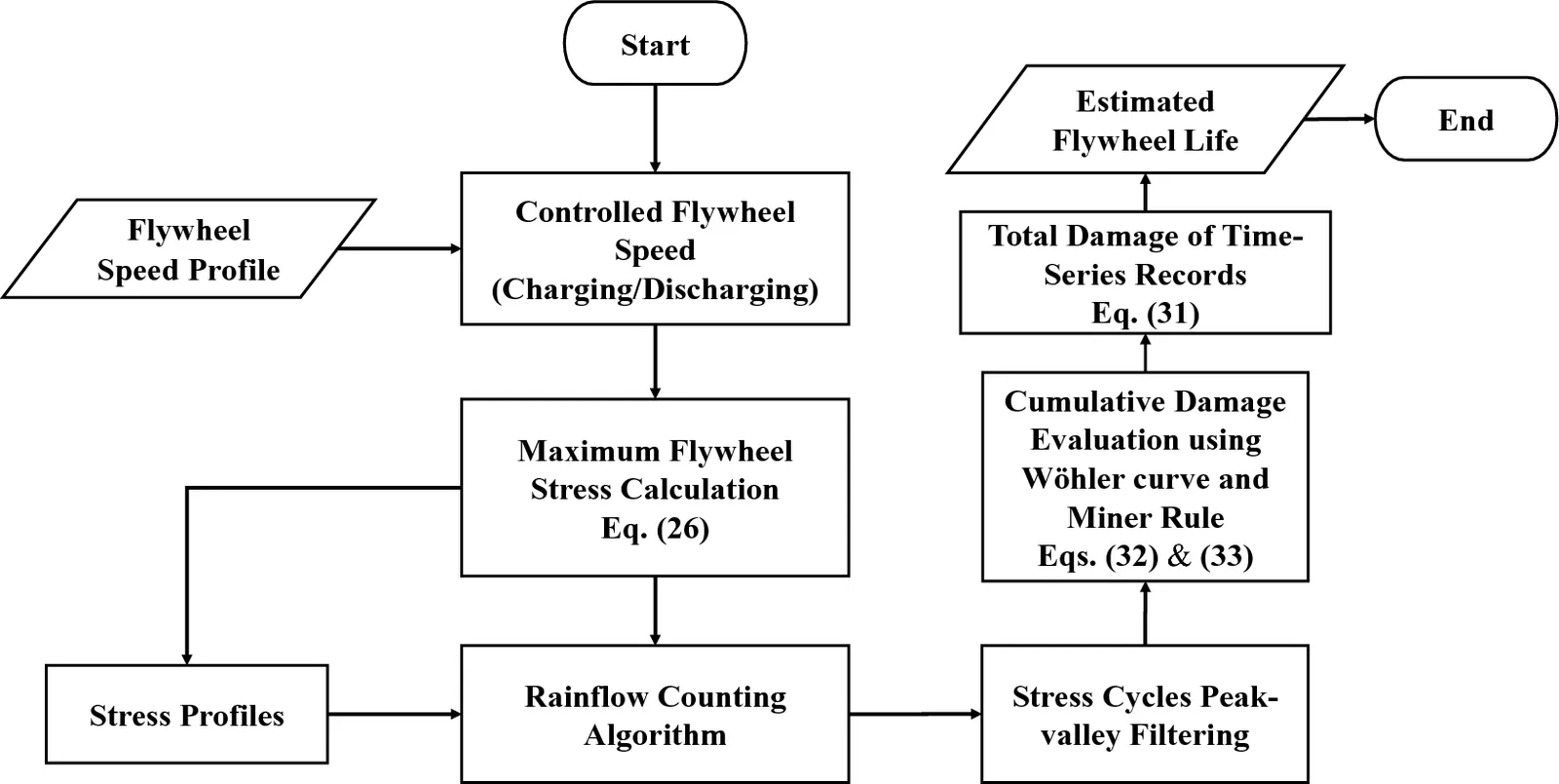
A flowchart illustrating the steps of the Fatigue Life algorithm.

In practice, lifetime fatigue damage for FESS is inferred from time-series records whose duration is much shorter than the design life. To estimate total damage, the damage-cycle counts obtained from these short records must be extrapolated over the full design lifetime. Accordingly, Eqs. (27) to (29) are reformulated to represent cumulative damage arising from one or more of such input time-series,

30 $${D}_{j}^{Life}=\sum_{i}\frac{{n}_{ji}^{Life}}{{N}_{ji}},$$

31 $${D}^{Life}=\sum_{j}{D}_{j}^{Life},$$

32 $${N}_{ji}={\left(\frac{{L}^{ult}-|{L}^{MF}|}{\frac{1}{2}{L}_{ji}^{RF}}\right)}^{m},$$

33 $${L}_{ji}^{RF}={L}_{ji}^{R}\left(\frac{{L}^{ult}-|{L}^{MF}|}{{L}^{ult}-|{L}_{ji}^{M}|}\right),$$

where ${D}_{j}^{\mathrm{L}\mathrm{i}\mathrm{f}\mathrm{e}}$ represents the extrapolated cumulative damage over the design lifetime associated with the ${j}^{\mathrm{t}\mathrm{h}}$ time series, ${n}_{ji}^{\mathrm{L}\mathrm{i}\mathrm{f}\mathrm{e}}$ denotes the extrapolated number of cycles, and ${N}_{ji}$ is the corresponding number of cycles to failure. The term ${L}_{ji}^{R}$ refers to the load range about the mean load ${L}_{ji}^{M}$ for the ${i}^{\mathrm{t}\mathrm{h}}$ cycle within the ${j}^{\mathrm{t}\mathrm{h}}$ time series. In Eqs. (27) to (33), *D* is the dimensionless cumulative Miner damage, ${n}_{i}$ is the number of counted occurrences of cycle *i*, and ${N}_{i}$ is the corresponding number of cycles to failure. The quantities ${L}_{i}^{R}$, ${L}_{i}^{RF}$, ${L}_{i}^{M}$, ${L}^{MF}$, and ${L}^{ult}$ are stress ranges or stress levels expressed in megapascals. Specifically, ${L}_{i}^{R}$ is the uncorrected rainflow stress range, ${L}_{i}^{M}$ is its mean stress, ${L}_{i}^{RF}$ is the mean-stress-corrected range, ${L}^{MF}$ is the selected fixed mean stress, and ${L}^{ult}$ is the material ultimate stress used in the Goodman correction. The Wöhler exponent *m* is dimensionless. The fatigue-life criterion is reached when the cumulative Miner damage equals unity (i.e., *D=1*). In our fatigue analysis algorithm developed in MATLAB as shown in Fig. 3, calculations can be carried out without applying Goodman correction. Under this condition, the algorithm assigns ${L}_{ji}^{RF}={L}_{ji}^{R}$ and uses ${L}^{MF}=0$ in Eq. (33). When this option is selected, the algorithm substitutes the value of ${L}^{ult}$ from S–N (Wöhler curve) inserted for the FESS material used^37^.

To estimate the fatigue-equivalent life from the finite simulated stress record, the rainflow cycles obtained from each time series are extrapolated according to the assumed long-term operating duty. For the ${j}^{th}$ simulated record, the counted number of occurrences of each stress cycle, ${n}_{ji}$, is multiplied by the ratio between the assumed service duration and the duration represented by the simulated record, giving the extrapolated cycle count ${n}_{ji}^{\mathrm{L}\mathrm{i}\mathrm{f}\mathrm{e}}$. The number of cycles to failure, ${N}_{ji}$, is then calculated from the mean-stress-corrected rainflow range ${L}_{ji}^{RF}$ using the adopted S–N relationship in Eqs. (32) and (33). The cumulative lifetime damage associated with the ${j}^{th}$ record is obtained from Eq. (30) and the contributions from all considered records are summed using Eq. (31). In the present analysis, the simulated charging/discharging profile is assumed to be representative and to repeat unchanged throughout the operating period. Under the Palmgren–Miner criterion, fatigue failure is assumed to occur when the accumulated damage reaches unity, ${D}^{\mathrm{L}\mathrm{i}\mathrm{f}\mathrm{e}}=1$. The corresponding fatigue-equivalent lifetime is therefore determined from the number of repeated simulated records, or equivalent operating years, required for cumulative damage to reach this limit.

## Control methodology

**Fig. 4:**
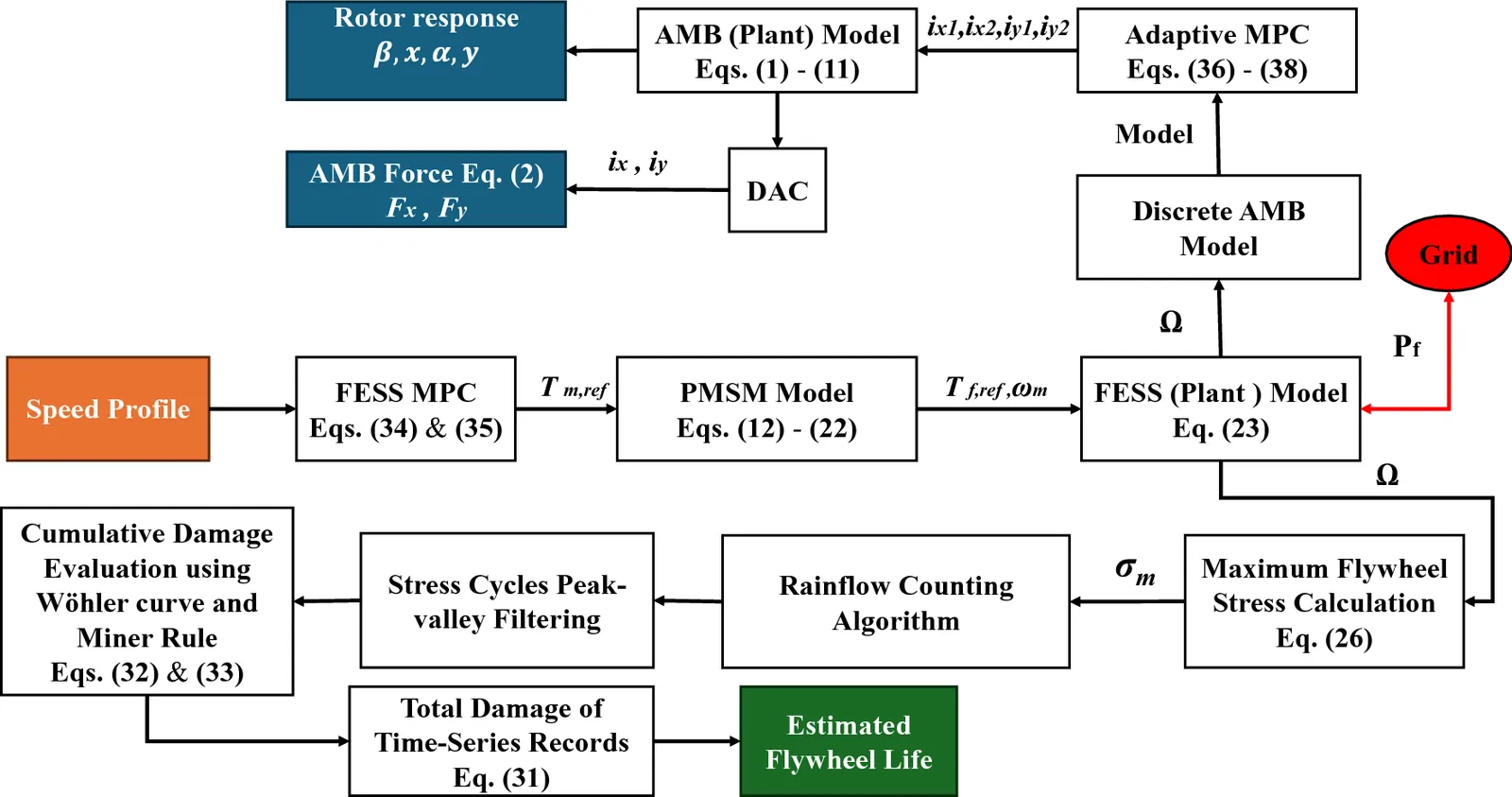
Integrated control-to-fatigue computational framework for the AMB-supported flywheel energy storage system.

The subsystem models described in Sections "System modeling" and "Fatigue damage assessment" are coupled sequentially at each simulation step, as shown in Fig. 4. First, the flywheel-speed reference is supplied to the FESS MPC, which calculates the required charge or discharge torque ${T}_{f}$. The PMSM model relates the commanded *d-q* currents to the electromagnetic torque ${T}_{e}$, which acts as the mechanical input to the flywheel model in Eq. (23). The updated mechanical speed *Ω* is then supplied to the AMB rotor model, where it modifies the gyroscopic matrix $\mathbf{G}(\Omega )$ and the mass-unbalance disturbance ${\mathbf{F}}_{unb}(\Omega ,t)$. The AMB controller calculates the four coil currents $\mathbf{i}$, and the rotor state-space model produces the translations, tilting angles, and sensor displacements included in ${\mathbf{x}}_{AMB}$ and ${\mathbf{y}}_{AMB}$. In parallel, the same speed history *Ω (t)* is converted through Eq. (26) into the nominal centrifugal-stress history ${\sigma}_{\mathrm{m}\mathrm{a}\mathrm{x}}(t)$. After completion of the simulated operating record, the stress turning points are extracted, rainflow cycles are counted, mean-stress correction is applied, and cumulative damage is calculated using the Palmgren–Miner rule. In the present model, the fatigue calculation is based on nominal centrifugal stress generated by the speed history. Local stresses associated with the shaft connection, geometric discontinuities, bearing-force application points, residual stress, and multiaxial loading are not included and are therefore identified as limitations of the current fatigue-life estimate.

**Fig. 5:**
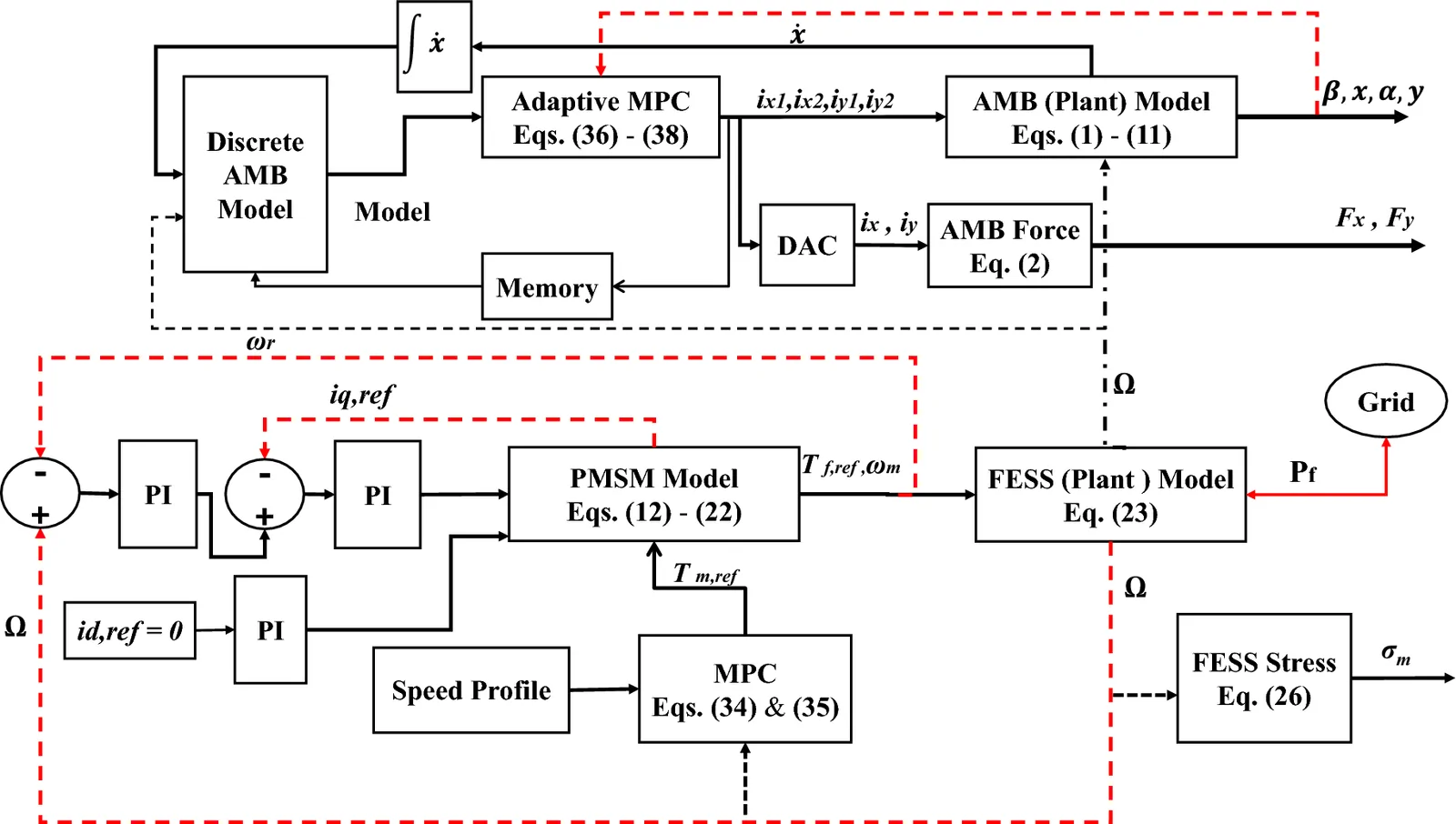
Integrated control system of the AMB-supported FESS with a PMSM drive using MPC and AMPC**.**

The control strategy for the integrated system, as shown in Fig. 5, uses a Model Predictive Controller (MPC) for the organized and adaptive operation of the FESS and the PMSM according to specific speed scenarios (i.e., charging and discharging scenarios). For the AMB system, an MPC is first designed to regulate the plant and ensure stable rotor dynamics. Once finalized, the MPC parameters are saved and used as the baseline controller. Next, an Adaptive Model Predictive Control (AMPC) algorithm^38,39^ is implemented to address time-varying behavior in the AMB system, most notably changes in flywheel speed, which modify the plant dynamics. During simulation, the AMPC updates the AMB model parameters in real-time, so the controller remains properly tuned as operating conditions evolve. The AMPC ability to update the state transition matrix is crucial for handling these variations. Additionally, a PI controller is embedded in the PMSM model to track and maintain the flywheel speed as a feedback measurement throughout the system’s operation. The MPC manages and controls both the FESS and PMSM to enhance the overall system’s responsiveness to dynamic conditions.

### Model predictive control of flywheel rotor speed

The MPC design for the FESS uses a sampling period of $\Delta t=0.2 \mathrm{s}$. The controller uses an ${N}_{p}=150$ steps prediction horizon to forecast system behavior and optimize performance over future steps. The control horizon is set to provide an actively optimized window of 1 s. With these settings, the controller solves a discretized optimal control problem equivalent represented by the following formulation aiming to reduce cyclic loading on the FESS rotor to mitigate fatigue damage and thereby increase rotor lifetime,

34 $$\begin{array}{c}\begin{array}{cc}\underset{{\mathbf{u}}_{1}(i)}{max}\&& {C}_{1}=\sum_{i=0}^{{{N}_{p}}_{1}-1}{F}_{1}\left({\mathbf{x}}_{1}(i),{\mathbf{u}}_{1}(i),{\mathbf{d}}_{1}(i)\right),\\ subject to\&& \begin{array}{c}{\mathbf{x}}_{1}\left(i+1\right)={\mathbf{A}}_{d1}\hspace{0.17em}{\mathbf{x}}_{1}\left(i\right)+{\mathbf{B}}_{d1}\hspace{0.17em}{\mathbf{u}}_{1}\left(i\right),\\ {\mathbf{x}}_{1}(0)={\mathbf{x}}_{\mathrm{1,0}},\\ {G}_{1}\left({\mathbf{x}}_{1}(i),{\mathbf{u}}_{1}(i),{\mathbf{d}}_{1}(i)\right)\ge 0.\end{array}\end{array} ,\\ \\ \\ \end{array}$$

where ${C}_{1}$ is cost function, ${\mathbf{u}}_{1}(i)$ is The control input vector at time step *i*, ${\mathbf{x}}_{1}(\mathrm{i})$ is the state vector at time step *i*, ${\mathbf{d}}_{1}(i)$ is the measured disturbance vector at time step *i*, ${F}_{1}(\cdot )$ is the stage cost function that defines the control objectives (e.g., tracking error, control effort) according to the equation reported in^40^, ${{N}_{p}}_{1}$ is the prediction horizon, ${\mathbf{A}}_{\mathbf{d}1}$ and ${\mathbf{B}}_{\mathbf{d}1}$ are the discrete-time state-space matrices of the plant model, ${\mathbf{x}}_{\mathrm{1,0}}$ is the initial states (typically the current measured or estimated state), and ${G}_{1}(\cdot )\ge 0$ represents the constraints on states, inputs, and outputs. The stage cost function is described as follows:

35 $$\begin{aligned}{F}_{1}({\mathbf{x}}_{1}(i),{\mathbf{u}}_{1}(i),{\mathbf{d}}_{1}(i))&={w}_{\Omega }\sum_{i=1}^{p}{\left(r({k}_{1}+i\mid {k}_{1})-\Omega ({k}_{1}+i\mid {k}_{1})\right)}^{2}\\&+{{w}_{u}}_{1}\sum_{i=0}^{p-1}{\left(\tau ({k}_{1}+i\mid {k}_{1})\right)}^{2}\\&+{{w}_{\Delta u}}_{1}\sum_{i=1}^{p-1}{\left(\tau ({k}_{1}+i\mid {k}_{1})-\tau ({k}_{1}+i-1\mid {k}_{1})\right)}^{2}\\&+{{\rho}_{e}}_{1}{{\epsilon}_{k}}_{1},\end{aligned}$$

where ${w}_{\Omega }$ is weight of speed output for reference tracking, ${{w}_{u}}_{1}$ is weight of control effort for flywheel input torque *τ*, ${{w}_{\Delta u}}_{1}$ is the weight of input smoothing rate to reduce sharp changes in the input torque, ${{\epsilon}_{k}}_{1}$ is slack variable at the control interval ${k}_{1}$ (dimensionless) and ${{\rho}_{e}}_{1}$ is constraint violation penalty weight (dimensionless).

### Adaptive model predictive control of active magnetic bearings (AMB)

The dynamic behavior of the AMB-supported rotor varies with the flywheel rotational speed because the gyroscopic terms in the rotor model depend explicitly on *Ω*. A conventional MPC Controller block uses a fixed prediction model specified at the controller-design stage and therefore does not directly account for this operating-point variation. In the present study, the AMB system is controlled using the Simulink Adaptive MPC Controller block, which permits the internal prediction model to be updated during simulation. Adaptation in this work refers specifically to the online update of the AMB state matrix according to the measured rotor speed; it does not refer to recursive identification of unknown physical parameters from input–output measurements. The scheduling variable at sampling instant *k* is defined as,

36-a $${\theta}_{k}={\Omega}_{k,}$$

where ${\Omega}_{k}$ is the measured mechanical rotor speed in $\mathrm{r}\mathrm{a}\mathrm{d}/\mathrm{s}$. The speed-dependent gyroscopic matrix is given by

36-b $$\mathbf{G}\left({\Omega}_{k}\right)=\left[\begin{array}{cccc}0& 0& {I}_{z}{\Omega}_{k}& 0\\ 0& 0& 0& 0\\ -{I}_{z}{\Omega}_{k}& 0& 0& 0\\ 0& 0& 0& 0\end{array}\right]$$

Using the AMB state vector,

$${\mathbf{x}}_{AMB}={\left[\begin{array}{cc}{\mathbf{q}}^{T}& {\dot{\mathbf{q}}}^{T}\end{array}\right]}^{T}\in {\mathbb{R}}^{8},$$

the continuous-time state matrix is reconstructed at each sampling instant as,

36-c $${\mathbf{A}}_{c,2}\left({\Omega}_{k}\right)=\left[\begin{array}{cc}{0}_{4\times 4}& {\mathbf{I}}_{4\times 4}\\ -{\mathbf{M}}^{-1}{\mathbf{K}}_{ss}& -{\mathbf{M}}^{-1}\mathbf{G}\left({\Omega}_{k}\right)\end{array}\right]\in {\mathbb{R}}^{8\times 8}.$$

The updated continuous-time matrix is converted to discrete time using the controller sampling interval ${T}_{s}=0.2{\hspace{0.17em}}\mathrm{s}$,

36-d $${\mathbf{A}}_{d,2}\left({\Omega}_{k}\right)=\mathrm{exp}{\hspace{0.17em}}\left[{\mathbf{A}}_{c,2}\left({\Omega}_{k}\right){T}_{s}\right]$$

The input, output, and direct-feedthrough matrices are retained at their nominal values because the force–current coefficients, rotor mass properties, bearing geometry, and displacement-sensor configuration are assumed to remain constant over the investigated operating range. Accordingly, the discrete prediction model used by the controller is

36-e $${\mathbf{x}}_{2}(i+1\mid k)={\mathbf{A}}_{d,2}({\Omega}_{k}){\mathbf{x}}_{2}(i\mid k)+{\mathbf{B}}_{d,2}{\mathbf{u}}_{2}(i\mid k)+{\mathbf{E}}_{d,2}{\mathbf{d}}_{2}(i\mid k)$$

36-f $${\mathbf{y}}_{2}(i\mid k)={\mathbf{C}}_{d,2}{\mathbf{x}}_{2}(i\mid k)+{\mathbf{D}}_{d,2}{\mathbf{u}}_{2}(i\mid k),$$

where ${\mathbf{x}}_{2}\in {\mathbb{R}}^{8}$ is the AMB rotor-state vector, ${\mathbf{u}}_{2}\in {\mathbb{R}}^{4}$ is the vector of bearing control currents, ${\mathbf{y}}_{2}\in {\mathbb{R}}^{4}$ is the measured displacement vector, and ${\mathbf{d}}_{2}$ represents the rotor-unbalance disturbance. The most recently measured value of ${\Omega}_{k}$ is assumed to remain constant over the current prediction horizon. The prediction model is updated again when a new rotor-speed measurement becomes available at the next sampling instant. At each control interval, the Adaptive MPC Controller solves the following constrained optimization problem:

37 $$\begin{array}{cc}subject to& \begin{array}{c}\underset{{\mathbf{u}}_{2}(i\mid k)}{\mathrm{m}\mathrm{i}\mathrm{n}}{C}_{2}(k)=\sum_{i=0}^{{N}_{p,2}-1}{F}_{2}\left[{\mathbf{x}}_{2}(i\mid k),{\mathbf{u}}_{2}(i\mid k),{\mathbf{d}}_{2}(i\mid k)\right],\\ {\mathbf{x}}_{2}(i+1\mid k)={\mathbf{A}}_{d,2}({\Omega}_{k}){\mathbf{x}}_{2}(i\mid k)+{\mathbf{B}}_{d,2}{\mathbf{u}}_{2}(i\mid k)+{\mathbf{E}}_{d,2}{\mathbf{d}}_{2}(i\mid k),\\ {\mathbf{u}}_{2,\mathrm{m}\mathrm{i}\mathrm{n}}\le {\mathbf{u}}_{2}(i\mid k)\le {\mathbf{u}}_{2,\mathrm{m}\mathrm{a}\mathrm{x}},\\ \Delta {\mathbf{u}}_{2,\mathrm{m}\mathrm{i}\mathrm{n}}\le \Delta {\mathbf{u}}_{2}(i\mid k)\le \Delta {\mathbf{u}}_{2,\mathrm{m}\mathrm{a}\mathrm{x}},\\ {\mathbf{y}}_{2,\mathrm{m}\mathrm{i}\mathrm{n}}-{{\boldsymbol{\epsilon}}}_{k}\le {\mathbf{y}}_{2}\left(i∣k\right)\le {\mathbf{y}}_{2,\mathrm{max}}+{{\boldsymbol{\epsilon}}}_{k},{{\boldsymbol{\epsilon}}}_{k}\ge 0.\end{array}\end{array}$$

where ${N}_{p,2}$ is the AMB prediction horizon, ${\widehat{\mathbf{x}}}_{2,k}$ is the state estimate at sampling instant *k*, and ${{{\epsilon}}}_{k}$ is a nonnegative slack variable used to soften the displacement constraints during severe transient conditions. The same current, current-rate, and displacement constraints are imposed after every model update. The stage cost function is described as follows:

38 $$\begin{aligned}{F}_{2}\left({\mathbf{x}}_{2}\left(i\right),{\mathbf{u}}_{2}\left(i\right),{\mathbf{d}}_{2}\left(i\right)\right)&={{\rho}_{e}}_{2}{{\epsilon}_{k}}_{2}+{w}_{\beta }{\sum}_{i=1}^{p}{\left[{r}_{\beta }\left({k}_{2}+i\mid {k}_{2}\right)-\beta \left({k}_{2}+i\mid {k}_{2}\right)\right]}^{2}\\&+{w}_{x}{\sum}_{i=1}^{p}{\left[{r}_{x}\left({k}_{2}+i\mid {k}_{2}\right)-x\left({k}_{2}+i\mid {k}_{2}\right)\right]}^{2}\\&+{w}_{\alpha }{\sum}_{i=1}^{p}{\left[{r}_{\alpha }({k}_{2}+i\mid {k}_{2})-\alpha ({k}_{2}+i\mid {k}_{2})\right]}^{2}\\&+{w}_{y}{\sum}_{i=1}^{p}{\left[{r}_{y}({k}_{2}+i\mid {k}_{2})-y({k}_{2}+i\mid {k}_{2})\right]}^{2}\\&+{{w}_{u}}_{2}{\sum}_{i=0}^{p-1}[({i}_{xA}({k}_{2}+i\mid {k}_{2}){)}^{2}+ ({i}_{xB}({k}_{2}+i\mid {k}_{2}){)}^{2}\\&+({i}_{yA}({k}_{2}+i\mid {k}_{2}){)}^{2}+({i}_{yB} ({k}_{2}+i\mid {k}_{2}){)}^{2}]\\&+({i}_{yA}({k}_{2}+i\mid {k}_{2}){)}^{2}+({i}_{yB} ({k}_{2}+i\mid {k}_{2}){)}^{2}]\\&+{{w}_{\Delta u}}_{2}{\sum}_{i=1}^{p-1}\left[{\left({i}_{xA}({k}_{2}+i\mid {k}_{2})-{i}_{xA} ({k}_{2}+i-1\mid {k}_{2})\right)}^{2}\right.\\ &\left.+{\left({i}_{xB}\left({k}_{2}+i\mid k_{2}\right)-{i}_{xB} ({k}_{2}+i-1\mid {k}_{2})\right)}^{2}\right.\\&+{ ({i}_{yA}({k}_{2}+i\mid {k}_{2})-{i}_{yA}({k}_{2}+i-1\mid {k}_{2}))}^{2}\\&+{({i}_{yB}({k}_{2}+i\mid {k}_{2})-{i}_{yB}({k}_{2}+i-1\mid {k}_{2}))}^{2}]\end{aligned}$$

where the cost function ${F}_{2}$ represents the adaptive MPC objective for AMB control by penalizing rotor tracking errors in the displacement and angular states *x*, *y*, *α*, and *β*. The weighting factors ${w}_{x}$, ${w}_{y}$, ${w}_{\alpha }$, and ${w}_{\beta }$ determine the relative importance of each tracking error. In addition, ${w}_{u2}$ limits excessive coil-current effort, while ${w}_{\Delta u2}$ promotes smooth current variation. The slack weights ${\rho}_{e2}$ and ${{\epsilon}_{k}}_{2}$ penalizes constraint relaxation during transient operation.

## Simulation results

**Fig. 6:**
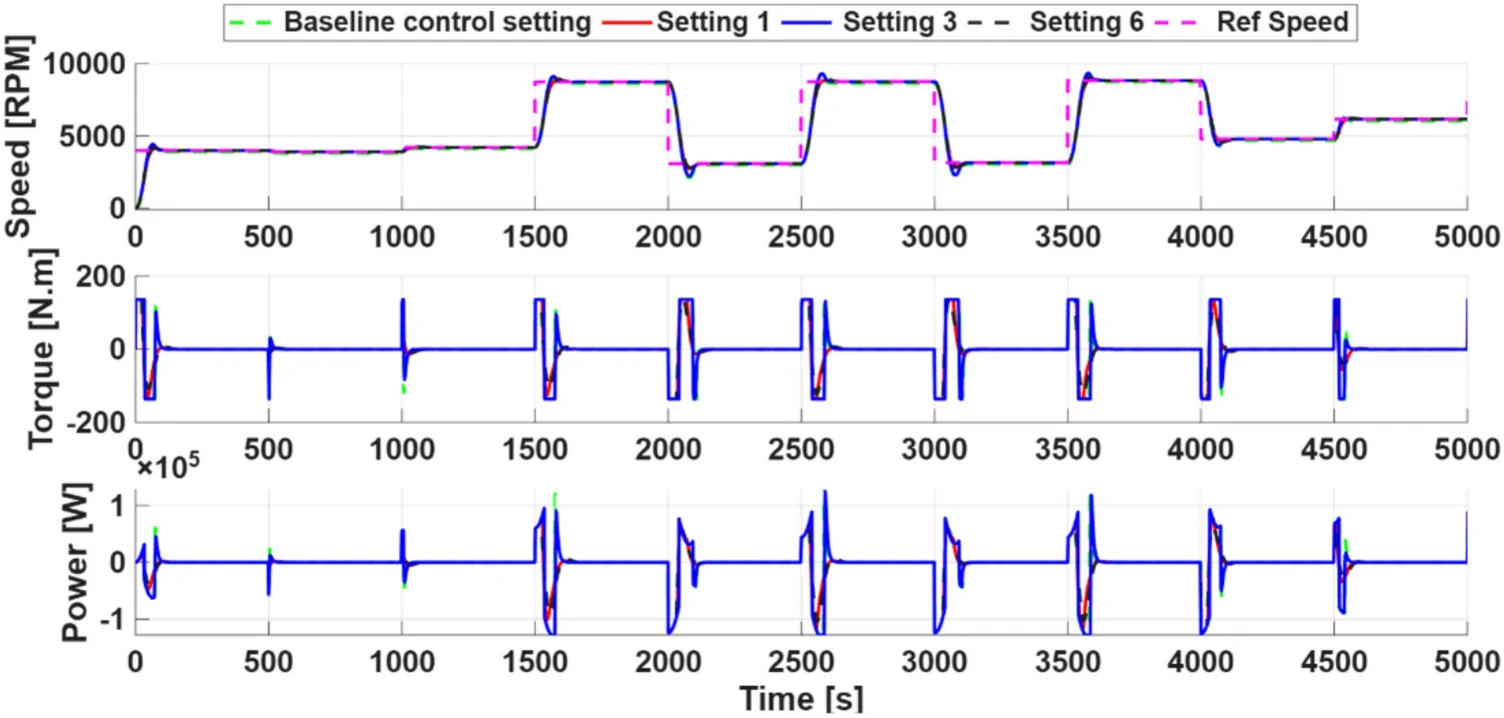
Comparison of power flow during charging and discharging for three different control settings and baseline control, according to the response of the applied speed profile and input control torque**.**

**Fig. 7:**
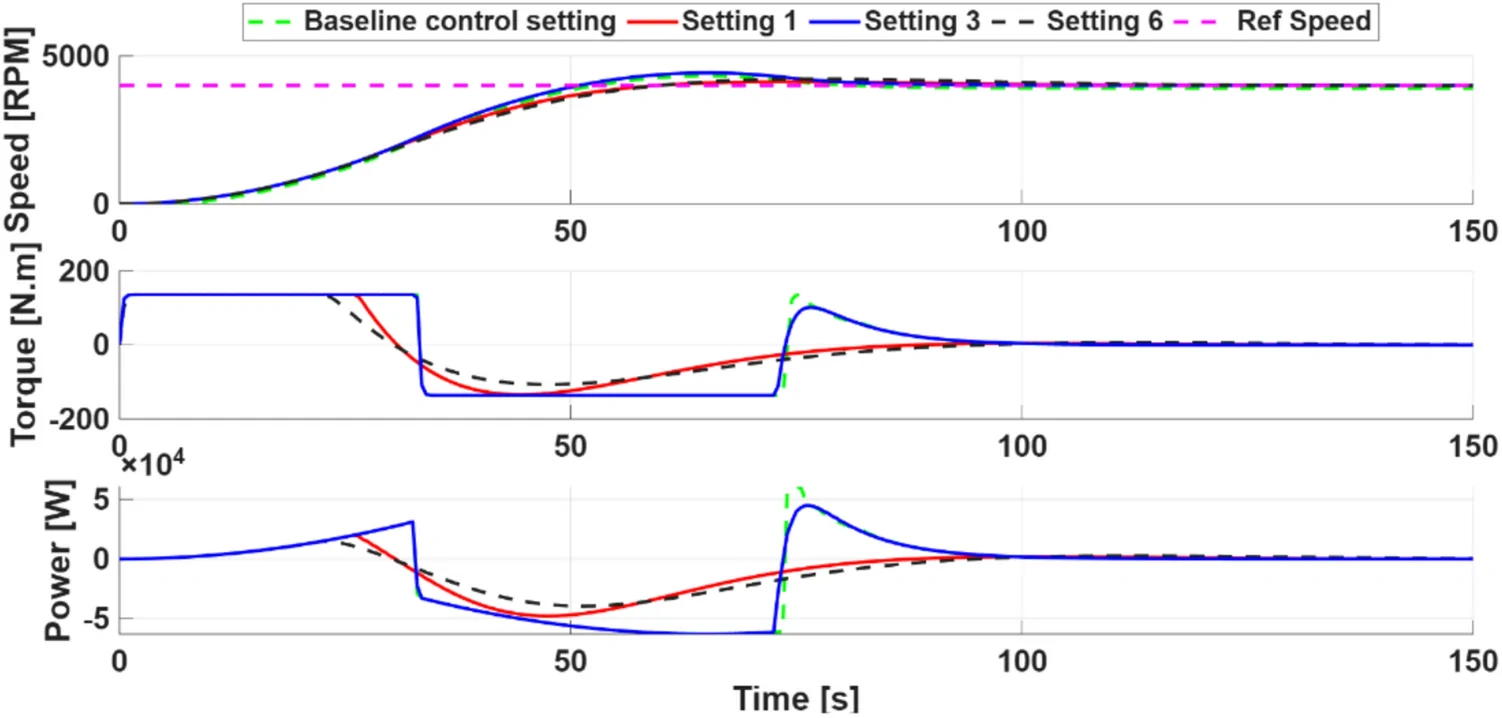
Comparison of power flow during charging and discharging for three different control settings and baseline control, according to the response of the applied speed profile and input control torque for zoomed transient view (150 s)**.**

**Fig. 8:**
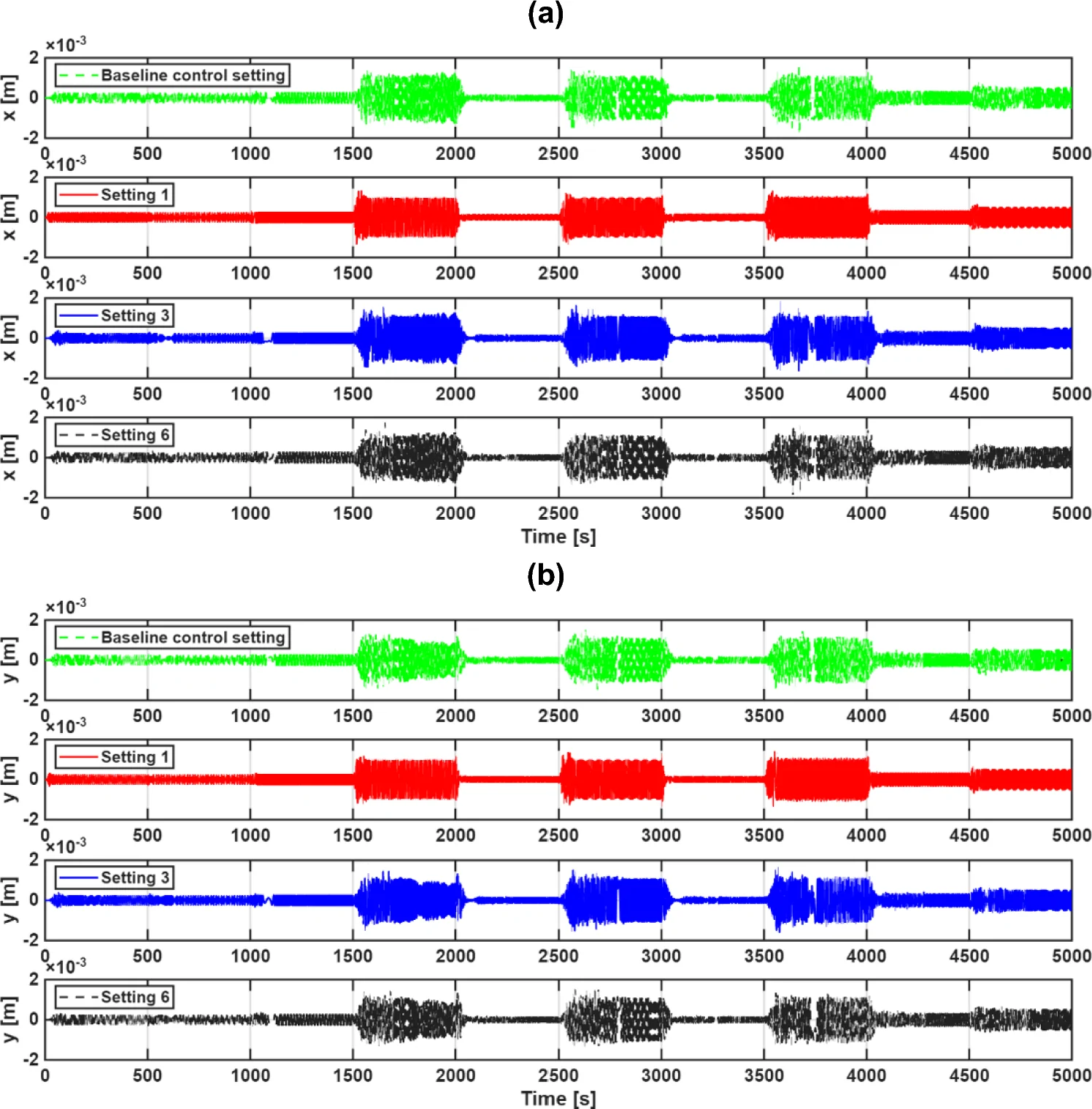
Rotor center displacement in *x* (**a**) and *y*(**b**) for variable speed profiles due to three different control settings and baseline control.

**Fig. 9:**
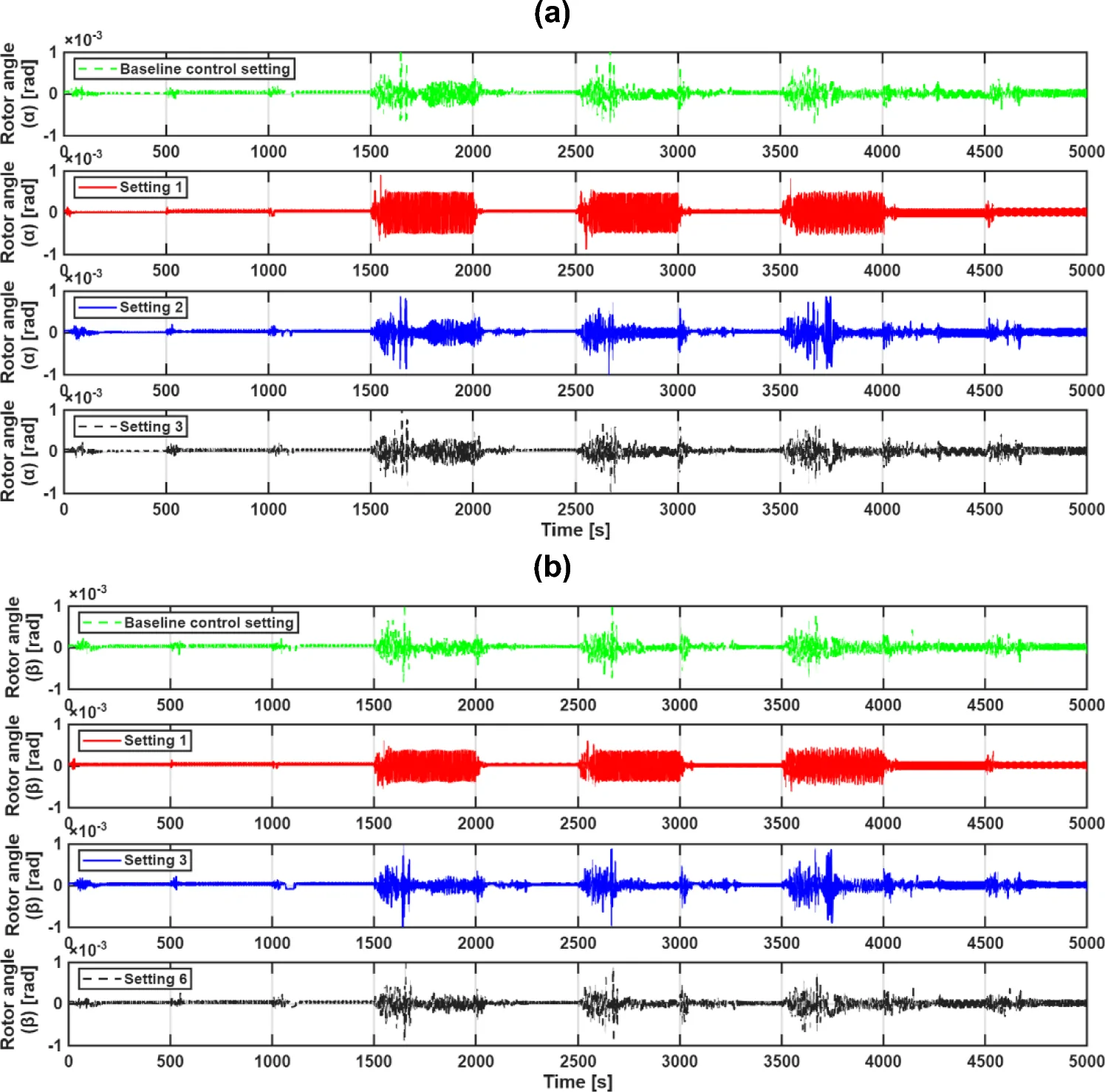
Rotor center angular displacement *α* (**a**) and *β* (**b**) for variable speed profiles due to three different control settings and baseline control.

**Fig. 10:**
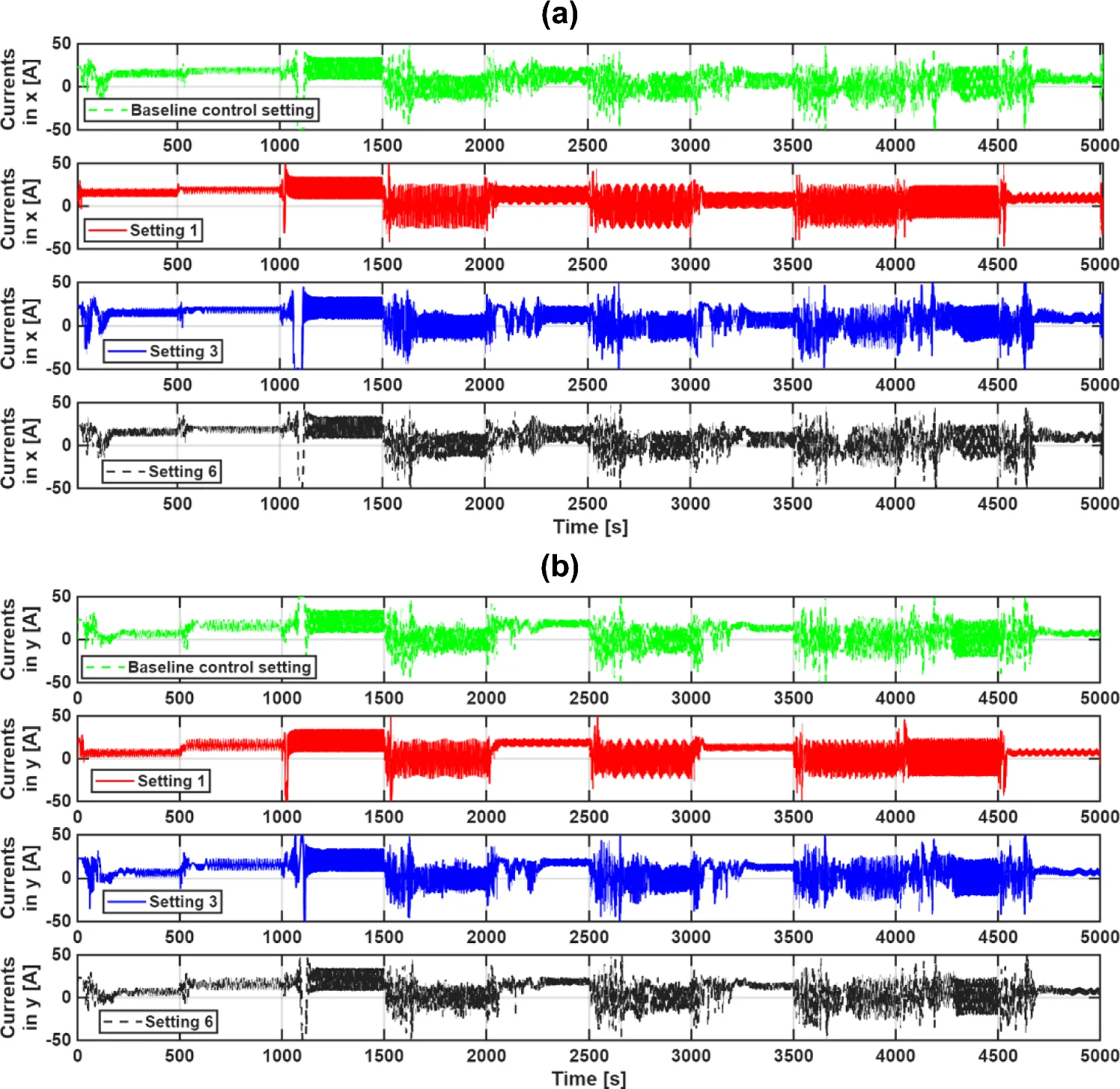
AMB coil currents in x (**a**) and $\mathrm{y}$(**b**) directions for variable speed profiles due to three different control settings and baseline control.

**Fig. 11:**
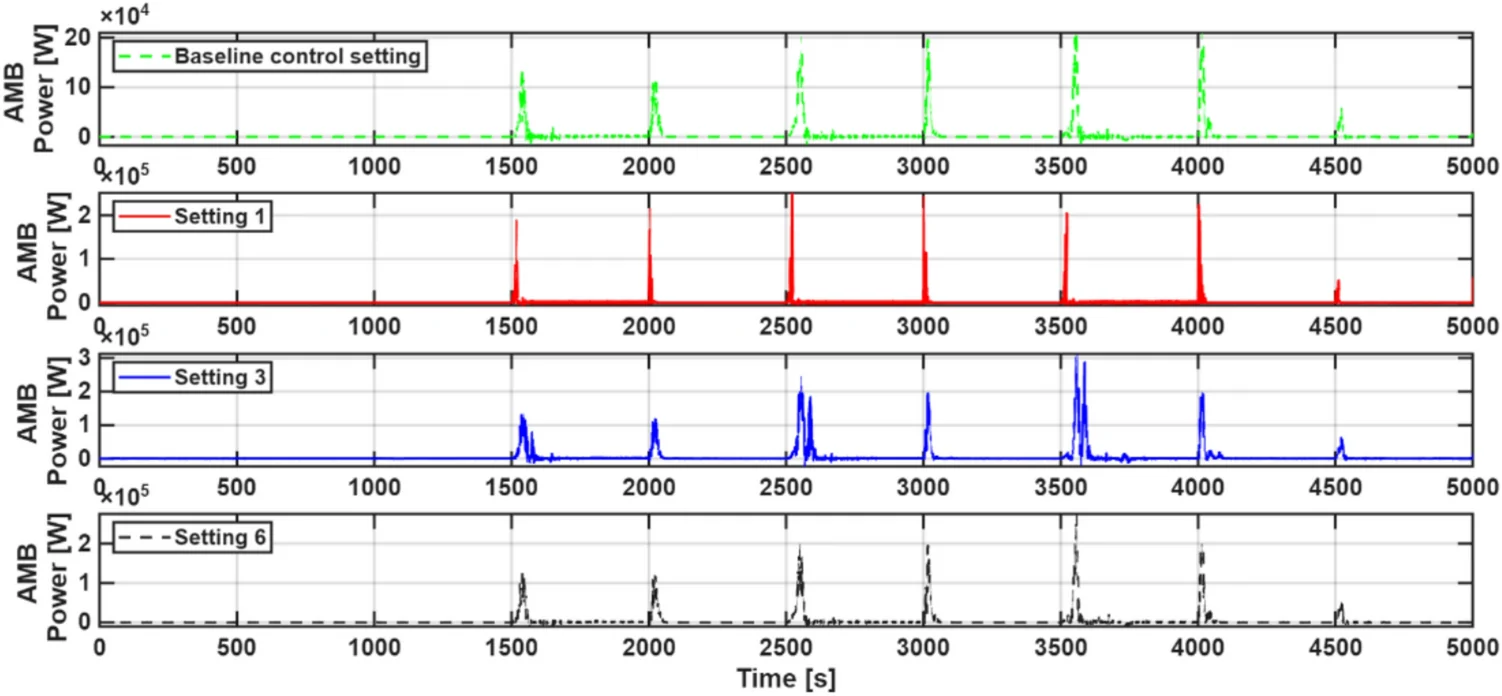
AMB power consumption for variable speed profiles due to three different control settings and baseline control.

**Fig. 12:**
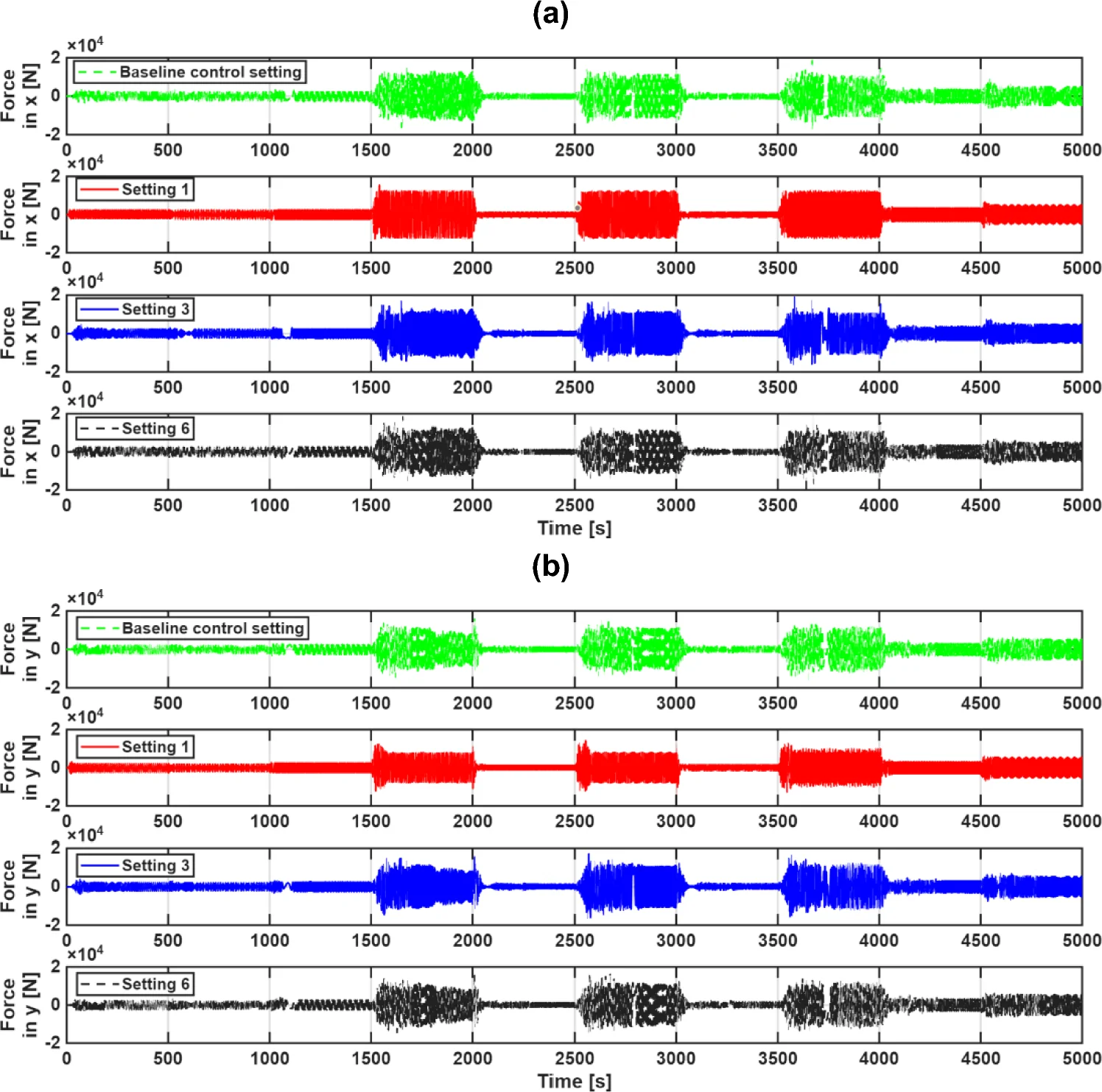
Magnetic force acting on rotor in x (**a**) and $\mathrm{y}$ (**b**) directions for variable speed profiles due to three different control settings and baseline control.

**Fig. 13:**
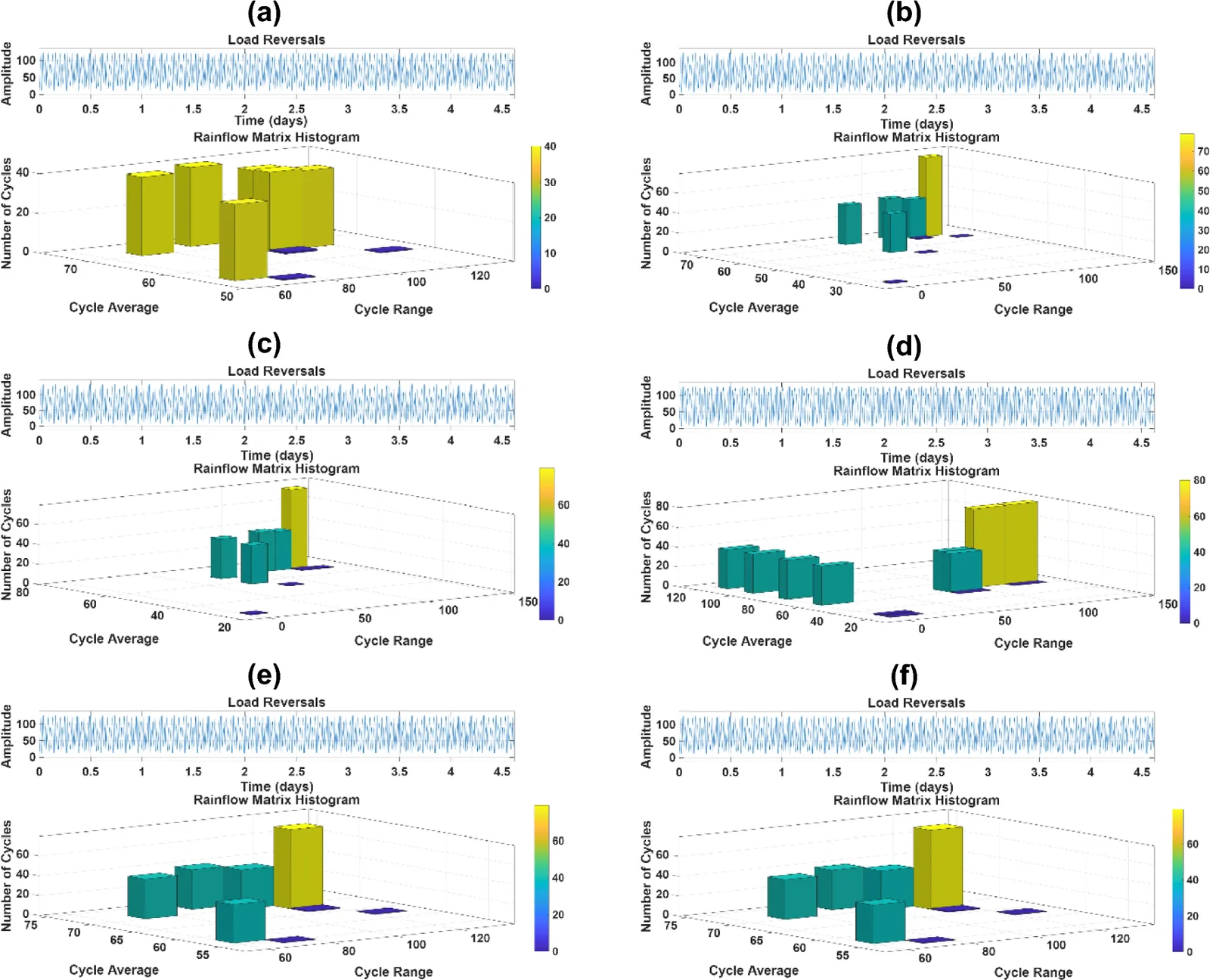
Load reversals and rainflow matrix histogram for control (**a**) Setting 1 (**b**) Setting 2 (**c**) Setting 3 (**d**) Setting 4 (**e**) Setting 5 (**f**) Setting 6.

The closed-loop behavior is assessed using simulated rotor translations, angular displacements, AMB currents, magnetic forces, and AMB power consumption. The bounded responses obtained during charging and discharging indicate satisfactory closed-loop operation over the investigated speed range. Table 3 summarizes the six MPC tuning cases investigated in this study and compares them with a baseline MPC controller that does not include fatigue-oriented weighting. The control-effort weight was fixed at ${w}_{u}=0.18$, whereas the input-smoothing weight ${w}_{\Delta u}$ and speed-reference tracking weight ${w}_{\Omega }$ were varied. Settings 1 to 3 employ a low input-smoothing penalty, with 5 and 10, respectively. Settings 4 to 6 use a substantially larger smoothing penalty, ${w}_{\Delta u}=100$, while retaining the same three tracking-weight values. This arrangement enables the effects of reference-tracking emphasis and torque-command smoothing to be evaluated independently. All cases were simulated for $4.00\times {10}^{5}$ s using $4.00\times {10}^{7}$ samples. For clarity, Fig. 6,7,8,9,10,11,12,13,14 compare the baseline controller with settings 1, 3, and 6, while the quantitative results for the baseline and all six MPC settings are summarized in Table 4,5,6,7.

**Table 3:** Different cases of control settings.

Control setting	Control effort weight, ${{{w}}}_{{{u}}}$	Input smoothing rate weight, ${{{w}}}_{{{\Delta}}{{u}}}$	Output reference tracking weight, ${{{w}}}_{\Omega }$
1st	0.18	0.1	1
2nd	0.18	0.1	5
3rd	0.18	0.1	10
4th	0.18	100	1
5th	0.18	100	5
6th	0.18	100	10

**Fig. 14:**
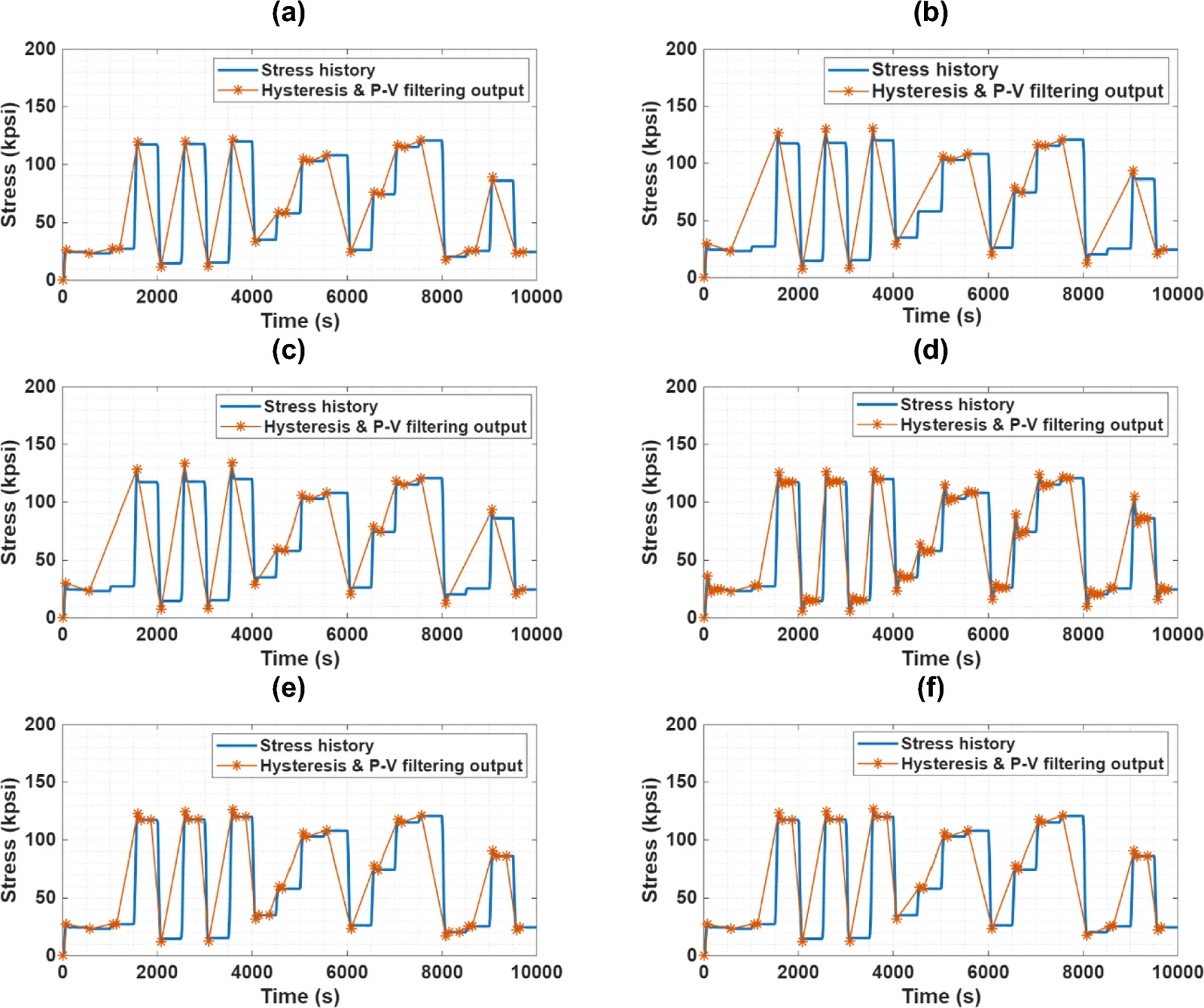
Peak-valley filtering for control (**a**) Setting 1 (**b**) Setting 2 (**c**) Setting 3 (**d**) Setting 4 (**e**) Setting 5 (**f**) Setting 6.

**Table 4:** Quantitative comparison of the dynamic performance of the baseline controller and the six MPC control settings—Simulation conditions and torque response.

Performance metric	Baseline	Setting 1	Setting 2	Setting 3	Setting 4	Setting 5	Setting 6
RMS torque, ${{{T}}}_{\mathbf{R}\mathbf{M}\mathbf{S}}$(N·m)	45.047	34.468	43.158	44.574	30.419	40.794	31.685

**Table 5:** Quantitative comparison of the dynamic performance of the baseline controller and the six MPC control settings—Rotor translational and angular response.

Performance metric	Baseline	Setting 1	Setting 2	Setting 3	Setting 4	Setting 5	Setting 6
Mean ${{x}}$-displacement, $\overline{{{x}} }$(m)	$1.240\times {10}^{-6}$	$5.690\times {10}^{-7}$	$6.600\times {10}^{-7}$	$1.090\times {10}^{-6}$	$1.190\times {10}^{-6}$	$7.570\times {10}^{-7}$	$1.140\times {10}^{-6}$
Mean ${{y}}$-displacement, $\overline{{{y}} }$(m)	$7.080\times {10}^{-7}$	$5.390\times {10}^{-7}$	$5.780\times {10}^{-7}$	$1.150\times {10}^{-6}$	$5.780\times {10}^{-7}$	$1.030\times {10}^{-6}$	$1.190\times {10}^{-6}$
RMS ${{x}}$-displacement, ${{{x}}}_{\mathbf{R}\mathbf{M}\mathbf{S}}$(m)	$4.640\times {10}^{-4}$	$4.460\times {10}^{-4}$	$4.650\times {10}^{-4}$	$4.670\times {10}^{-4}$	$4.640\times {10}^{-4}$	$4.510\times {10}^{-4}$	$4.640\times {10}^{-4}$
RMS ${{y}}$-displacement, ${{{y}}}_{\mathbf{R}\mathbf{M}\mathbf{S}}$(m)	$4.640\times {10}^{-4}$	$4.570\times {10}^{-4}$	$4.650\times {10}^{-4}$	$4.670\times {10}^{-4}$	$4.640\times {10}^{-4}$	$4.790\times {10}^{-4}$	$4.640\times {10}^{-4}$
Peak-to-peak ${{x}}$-displacement, ${{{x}}}_{\mathbf{p}-\mathbf{p}}$(m)	$4.197\times {10}^{-3}$	$3.430\times {10}^{-3}$	$4.500\times {10}^{-3}$	$4.020\times {10}^{-3}$	$4.420\times {10}^{-3}$	$3.360\times {10}^{-3}$	$4.050\times {10}^{-3}$
Peak-to-peak ${{y}}$-displacement, ${{{y}}}_{\mathbf{p}-\mathbf{p}}$(m)	$3.990\times {10}^{-3}$	$3.630\times {10}^{-3}$	$4.140\times {10}^{-3}$	$4.010\times {10}^{-3}$	$4.140\times {10}^{-3}$	$3.720\times {10}^{-3}$	$3.830\times {10}^{-3}$
Peak resultant displacement, ${{{r}}}_{\mathbf{p}\mathbf{e}\mathbf{a}\mathbf{k}}$(m)	$2.186\times {10}^{-3}$	$1.884\times {10}^{-3}$	$2.395\times {10}^{-3}$	$2.079\times {10}^{-3}$	$2.240\times {10}^{-3}$	$1.880\times {10}^{-3}$	$2.109\times {10}^{-3}$
RMS resultant displacement, ${{{r}}}_{\mathbf{R}\mathbf{M}\mathbf{S}}$(m)	$6.565\times {10}^{-4}$	$6.390\times {10}^{-4}$	$6.572\times {10}^{-4}$	$6.603\times {10}^{-4}$	$6.564\times {10}^{-4}$	$6.583\times {10}^{-4}$	$6.561\times {10}^{-4}$
RMS ${{\alpha}}$-tilt, ${{{\alpha}}}_{\mathbf{R}\mathbf{M}\mathbf{S}}$(rad)	$1.153\times {10}^{-4}$	$1.720\times {10}^{-4}$	$1.160\times {10}^{-4}$	$1.193\times {10}^{-4}$	$1.160\times {10}^{-4}$	$2.350\times {10}^{-4}$	$1.160\times {10}^{-4}$
RMS ${{\beta}}$-tilt, ${{{\beta}}}_{\mathbf{R}\mathbf{M}\mathbf{S}}$(rad)	$1.161\times {10}^{-4}$	$1.440\times {10}^{-4}$	$1.160\times {10}^{-4}$	$1.196\times {10}^{-4}$	$1.160\times {10}^{-4}$	$1.870\times {10}^{-4}$	$1.160\times {10}^{-4}$
RMS resultant tilt, ${{{\theta}}}_{\mathbf{R}\mathbf{M}\mathbf{S}}$(rad)	$1.618\times {10}^{-4}$	$2.220\times {10}^{-4}$	$1.630\times {10}^{-4}$	$1.672\times {10}^{-4}$	$1.620\times {10}^{-4}$	$2.990\times {10}^{-4}$	$1.620\times {10}^{-4}$
Peak resultant tilt, ${{{\theta}}}_{\mathbf{p}\mathbf{e}\mathbf{a}\mathbf{k}}$(rad)	$1.541\times {10}^{-3}$	$1.120\times {10}^{-3}$	$1.570\times {10}^{-3}$	$1.549\times {10}^{-3}$	$1.570\times {10}^{-3}$	$1.100\times {10}^{-3}$	$1.570\times {10}^{-3}$

**Table 6:** Quantitative comparison of the dynamic performance of the baseline controller and the six MPC control settings—AMB current demand and power consumption.

Performance metric	Baseline	Setting 1	Setting 2	Setting 3	Setting 4	Setting 5	Setting 6
RMS ${{x}}$-axis current, ${{{i}}}_{{{x}},\mathbf{R}\mathbf{M}\mathbf{S}}$(A)	*14.996*	*14.421*	*15.151*	*15.098*	*15.094*	*14.345*	*14.968*
Peak ${{x}}$-axis current, ${{{i}}}_{{{x}},\mathbf{p}\mathbf{e}\mathbf{a}\mathbf{k}}$(A)	*50.000*	*49.508*	*50.000*	*50.000*	*50.000*	*50.000*	*50.000*
RMS ${{y}}$-axis current, ${{{i}}}_{{{y}},\mathbf{R}\mathbf{M}\mathbf{S}}$(A)	*14.886*	*14.670*	*15.090*	*14.964*	*15.090*	*14.793*	*14.969*
Peak ${{y}}$-axis current, ${{{i}}}_{{{y}},\mathbf{p}\mathbf{e}\mathbf{a}\mathbf{k}}$(A)	*50.000*	*50.000*	*50.000*	*50.000*	*50.000*	*50.000*	*50.000*
Peak resultant AMB current, ${{{i}}}_{\mathbf{A}\mathbf{M}\mathbf{B},\mathbf{p}\mathbf{e}\mathbf{a}\mathbf{k}}$(A)	*70.710*	*65.054*	*70.699*	*70.711*	*70.711*	*70.660*	*70.711*
RMS resultant AMB current, ${{{i}}}_{\mathbf{A}\mathbf{M}\mathbf{B},\mathbf{R}\mathbf{M}\mathbf{S}}$(A)	*21.130*	*20.571*	*21.360*	*21.257*	*21.343*	*20.606*	*21.169*
Mean AMB power consumption, ${\overline{{{P}}} }_{\mathbf{A}\mathbf{M}\mathbf{B}}$(W)	*3718.7*	*1791.1*	*3999.8*	*4741.5*	*3622.9*	*3482.3*	*3603.1*

**Table 7:** Quantitative comparison of the dynamic performance of the baseline controller and the six MPC control settings—Active magnetic bearing force response.

Performance metric	Baseline	Setting 1	Setting 2	Setting 3	Setting 4	Setting 5	Setting 6
RMS ${{x}}$-direction force, ${{{F}}}_{{{x}},\mathbf{R}\mathbf{M}\mathbf{S}}$(N)	*4707.2*	*4650.4*	*4711.9*	*4736.2*	*4705.2*	*4723.1*	*4701.2*
Peak ${{x}}$-direction force, ${{{F}}}_{{{x}},\mathbf{p}\mathbf{e}\mathbf{a}\mathbf{k}}$(N)	$\mathrm{22,525}$	$\mathrm{18,021}$	$\mathrm{24,548}$	$\mathrm{21,914}$	$\mathrm{22,894}$	$\mathrm{18,917}$	$\mathrm{21,770}$
RMS ${{y}}$-direction force, ${{{F}}}_{{{y}},\mathbf{R}\mathbf{M}\mathbf{S}}$(N)	*4652.6*	*4521.2*	*4656.5*	*4681.6*	*4650.3*	*4835.0*	*4649.1*
Peak ${{y}}$-direction force, ${{{F}}}_{{{y}},\mathbf{p}\mathbf{e}\mathbf{a}\mathbf{k}}$ (N)	$\mathrm{23,012}$	$\mathrm{17,955}$	$\mathrm{21,844}$	$\mathrm{21,272}$	$\mathrm{21,844}$	$\mathrm{18,576}$	$\mathrm{20,876}$
Peak resultant magnetic force, ${{{F}}}_{\mathbf{A}\mathbf{M}\mathbf{B},\mathbf{p}\mathbf{e}\mathbf{a}\mathbf{k}}$ (N)	$\mathrm{24,794}$	$\mathrm{20,460}$	$\mathrm{26,666}$	$\mathrm{25,725}$	$\mathrm{24,017}$	$\mathrm{19,915}$	$\mathrm{24,133}$
RMS resultant magnetic force, ${{{F}}}_{\mathbf{A}\mathbf{M}\mathbf{B},\mathbf{R}\mathbf{M}\mathbf{S}}$ (N)	*6618.5*	*6485.9*	*6615.5*	*6659.5*	*6615.5*	*6759.1*	*6611.7*

The two input-smoothing weights, ${w}_{\Delta u}=0.1$ and ${w}_{\Delta u}=100$, were deliberately selected to represent two limiting control behaviors rather than being obtained from a continuous sensitivity analysis. The lower value permits rapid control-input variations and emphasizes tracking performance, whereas the higher value strongly suppresses input-rate changes and promotes smoother actuation. Combined with the three tracking weights, these values were used to bound the trade-off between tracking aggressiveness, control smoothness, and fatigue-related performance. Therefore, the selected weights represent comparative boundary cases and are not claimed to be globally optimal. Figure 6 shows the flywheel speed, torque, and power responses over the complete operating profile, while Fig. 7 provides an enlarged view of the initial transient.

All controllers reproduce the sequence of charging, standby, and discharging operations, with the largest torque and power excursions occurring when the speed reference changes abruptly. The torque reverses sign between acceleration and deceleration, and the corresponding power response follows the direction of energy exchange between the flywheel and the grid. The peak torque is constrained to $136 \, \mathrm{N}\mathrm{.}\mathrm{m}$ for every case; consequently, the principal differences among the controllers are reflected in the duration, smoothness, and RMS magnitude of the torque command rather than its maximum value.

The enlarged transient in Fig. 7 illustrates the effect of the cost-function weights. Setting 3, which combines a low smoothing penalty with the highest tracking weight, produces comparatively rapid torque corrections and pronounced power transients. Setting 6 retains the same tracking weight but increases ${w}_{\Delta u}$ from 0.1 to 100, thereby smoothing the torque command and reducing its RMS value, although at the expense of a longer transient. Setting 1 provides the smallest speed overshoot among all investigated cases, equal to $3.23\mathrm{\%}$, compared with $10.66\mathrm{\%}$ for the baseline. Settings 5 and 6 also reduce the overshoot to $5.75\mathrm{\%}$ and $5.50\mathrm{\%}$, respectively. In contrast, setting 4 produces the largest overshoot, $22.12\mathrm{\%}$, and the longest settling time, *182.91* s. This result indicates that a large input-rate penalty does not necessarily improve the complete transient response when the tracking weight is too low.

The RMS torque values in Table 4 provide a complementary measure of actuation severity. Setting 4 gives the lowest RMS torque, 30.419 $\mathrm{N}.\mathrm{m}$, followed by setting 6 at $31.685 \, \mathrm{N}.\mathrm{m}$ and setting 1 at 34.468 $\mathrm{N}.\mathrm{m}$, compared with 45.047 $\mathrm{N}.\mathrm{m}$ for the baseline. These values correspond to reductions of approximately $32.5\mathrm{\%}$, $29.7\mathrm{\%}$, and $23.5\mathrm{\%}$, respectively. However, the low RMS torque of setting 4 is accompanied by excessive overshoot and a settling time more than twice that of the baseline. Therefore, RMS torque alone is insufficient for selecting the preferred controller; tracking accuracy, transient duration, and mechanical response must also be considered. Figure 8 shows the rotor-center displacements in the *x*- and *y*-directions. The displacement amplitudes increase during the high-speed intervals and immediately following speed transitions because the rotor unbalance force and gyroscopic effects depend on rotational speed. Nevertheless, the AMB controller maintains bounded rotor motion for all displayed cases throughout the complete operating profile.

The quantitative results in Table 5 show that setting 1 reduces the peak resultant rotor displacement from $2.186\times {10}^{-3}$ m for the baseline to $1.884\times {10}^{-3}$ m, representing a reduction of approximately $13.8\mathrm{\%}$. It also gives the lowest RMS resultant displacement, $6.390\times {10}^{-4}$ m, compared with $6.565\times {10}^{-4}$ m for the baseline. Setting 5 produces the lowest peak resultant displacement, $1.880\times {10}^{-3}$ m, and the smallest peak-to-peak *x*-displacement, $3.360\times {10}^{-3}$ m. However, its RMS *y*-displacement increases to $4.790\times {10}^{-4}$ m, the largest value among the tested settings. Setting 2 gives the largest peak resultant displacement, $2.395\times {10}^{-3}$ m, whereas settings 3, 4, and 6 remain close to the baseline value. These results show that peak and RMS displacement metrics do not necessarily vary in the same manner. A controller may suppress isolated displacement peaks while maintaining oscillations over a longer interval, leading to a comparatively high RMS value. Consequently, both measures are required to characterize AMB levitation quality under repeated speed changes.

The rotor angular responses *α* and *β* are presented in Fig. 9 and their quantitative results are summarized in Table 5. As with the translational response, the angular oscillations become more pronounced during acceleration, deceleration, and high-speed operation. The adaptive AMB controller maintains bounded angular motion in all cases, although the magnitude and persistence of the oscillations depend on the FESS-MPC tuning. Setting 5 gives the lowest peak resultant rotor tilt, $1.100\times {10}^{-3}$ rad, compared with $1.541\times {10}^{-3}$ rad for the baseline, corresponding to a reduction of approximately $28.6\mathrm{\%}$. Setting 1 similarly reduces the peak resultant tilt to $1.120\times {10}^{-3}$ rad. However, their RMS angular responses are higher than the baseline. The resultant RMS tilt increases from $1.618\times {10}^{-4}$ rad for the baseline to $2.220\times {10}^{-4}$ rad for setting 1 and $2.990\times {10}^{-4}$ rad for setting 5. In particular, setting 5 reduces the maximum angular excursion but produces more sustained angular activity, as reflected by its $84.8\mathrm{\%}$ increase in RMS resultant tilt relative to the baseline. Settings 2, 4, and 6 provide RMS resultant tilts of approximately $1.62\times {10}^{-4}$ rad, which are close to the baseline, although their peak values reach $1.570\times {10}^{-3}$ rad. Setting 3 produces a moderate increase in both RMS and peak angular response. The comparison confirms that minimizing peak rotor tilt does not necessarily minimize the energy or persistence of the angular oscillations.

Figure 10 presents the AMB coil-current responses in the *x*- and *y*-directions, while Fig. 11 shows the AMB power consumption. The corresponding quantitative results are summarized in Table 6. The current demand increases during the high-speed portions of the speed profile and during abrupt transitions, when larger magnetic forces are required to counteract the rotor disturbance. The individual currents approach the imposed *50* A limit in most cases. The resultant peak current is therefore approximately *70.71* A when both directional components simultaneously approach their limits. Setting 1 provides the lowest electrical demand among the investigated cases. Its resultant peak current is *65.054* A, approximately $8.0\mathrm{\%}$ below the baseline value of *70.710* A, while its RMS resultant current decreases from *21.130* to *20.571* A. More importantly, its mean AMB power consumption decreases from *3718.7* W for the baseline to *1791.1* W, corresponding to a reduction of approximately $51.8\mathrm{\%}$. Setting 5 also provides a relatively low RMS resultant current of *20.606* A and reduces mean power consumption to *3482.3* W. In contrast, setting 3 produces the highest mean-valued AMB power consumption, *4741.5* W, which is approximately $27.5\mathrm{\%}$ above the baseline. This increased demand is consistent with the rapid corrections associated with its high tracking weight and low input-smoothing penalty. Setting 2 also increases the mean AMB power consumption to *3999.8* W, whereas settings 4 and 6 produce values slightly below the baseline. These results demonstrate that a controller that achieves acceptable speed tracking may nevertheless impose a significantly larger energy demand on the magnetic-bearing system. Figure 12 shows the magnetic forces acting in the *x*- and *y*-directions. The corresponding quantitative results are summarized in Table 7. Their time-domain patterns correspond closely to the rotor displacement and current responses, with the largest amplitudes occurring during high-speed operation and immediately after reference changes.

Table 7 shows that setting 5 produces the lowest peak resultant magnetic force, $1.9915\times {10}^{4}$ N, representing a reduction of approximately $19.7\mathrm{\%}$ relative to the baseline value of $2.4794\times {10}^{4}$ N. Setting 1 gives the second-lowest peak value, $2.0460\times {10}^{4}$ N, corresponding to a $17.5\mathrm{\%}$ reduction. Despite its low peak force, setting 5 produces the largest RMS resultant magnetic force, $6.7591\times {10}^{3}$ N. This indicates that the controller distributes its force demand over a longer period rather than producing isolated high peaks. Setting 1, by comparison, gives both a reduced peak force and the lowest RMS resultant force, $6.4859\times {10}^{3}$ N. Setting 2 produces the highest peak resultant magnetic force, $2.6666\times {10}^{4}$ N, while setting 3 gives the second-highest value, $2.5725\times {10}^{4}$ N. Settings 4 and 6 remain slightly below the baseline peak force. The combined current, power, and force results show that setting 1 provides the most consistent reduction in AMB actuation demand. Setting 5 effectively suppresses force and displacement peaks, but its higher RMS force and angular response indicate more persistent corrective activity. Settings 2 and 3 impose the greatest peak or average bearing demands, whereas settings 4 and 6 provide intermediate behavior.

Table 7 demonstrates that no single setting minimizes every performance index. Setting 4 gives the lowest RMS torque, but its $22.12\mathrm{\%}$ overshoot and *182.91* s settling time (listed in Table 8) makes it unsuitable when rapid and safe speed regulation is required. Setting 5 minimizes peak magnetic force, peak rotor displacement, and peak rotor tilt, but produces the highest RMS resultant magnetic force and RMS rotor tilt. Setting 6 combines low RMS torque with moderate overshoot, acceptable AMB power consumption, and rotor responses close to the baseline. Settings 2 and 3 provide limited improvement over the baseline and generally increase AMB force, power demand, or fatigue damage. Among the evaluated cases, setting 1 provides the most balanced dynamic response. Relative to the baseline, it reduces speed overshoot by approximately $69.7\mathrm{\%}$, RMS torque by $23.5\mathrm{\%}$, peak resultant magnetic force by $17.5\mathrm{\%}$, peak resultant rotor displacement by $13.8\mathrm{\%}$, peak resultant AMB current by $8.0\mathrm{\%}$, and mean AMB power consumption by $51.8\mathrm{\%}$. These improvements are accompanied by an $8.6\mathrm{\%}$ increase in settling time and a higher RMS angular response. Therefore, the advantage of setting 1 lies in its combined reduction of speed overshoot, bearing effort, displacement peaks, and energy consumption rather than uniform improvement in every rotor-motion metric.

**Table 8:** Estimated FESS lifetime for different control settings.

Control setting	Overshoot percentage	Settling time (sec)	Steady-state error %	No. of cycles for time series	Cumulative damage for time series	No. of cycles	FESS lifetime (years)
Baseline	10.66	82.94	0.000004	446	1.83 e^−4^	2.44 e^6^	69.27
1st	3.23	90.06	0.000013	552	2.284 e^−5^	2.3e^7^	555.43
2nd	10.6	84.3	0.000007	405	1.922e^−4^	2.1e^6^	65.99
3rd	10.73	83.44	0.000014	443	2.628 e^−4^	1.69e^6^	48.27
4th	22.12	182.91	0.002579	1165	1.703 e^−4^	8.73e^6^	74.49
5th	5.75	108.35	0.000017	764	6.200 e^−5^	1.23e^7^	204.58
6th	5.5	105.35	0.000003	684	6.40 e^−5^	1.06 e^7^	198.11

The fatigue-life analysis was performed using the stress history generated from the controller-dependent flywheel-speed response. The complete simulated record has a duration of $4.00\times {10}^{5}$ s, equivalent to approximately *4.63* days. Peak–valley filtering was first applied to retain the stress reversals relevant to fatigue, after which the rainflow algorithm classified the cycles according to their ranges and mean values. Figure 13 presents the load reversals and rainflow matrices, while Fig. 14 compares the original stress histories with the retained peak–valley sequences.

The rainflow distributions demonstrate that fatigue severity cannot be inferred from cycle count alone. For example, setting 4 produces the largest number of counted cycles in the simulated record, 1165, but its cumulative damage remains lower than that of settings 2 and 3. Conversely, setting 3 contains 443 counted cycles yet produces the highest cumulative damage. This difference arises because the damage contribution of each cycle depends strongly on its stress range, mean stress, and corresponding cycles to failure on the S–N curve. High-amplitude cycles can therefore dominate cumulative damage even when their number is comparatively small. The low input-smoothing penalty used in settings 2 and 3 permits stronger torque corrections as the tracking weight increases. The resulting speed and stress transients shift the rainflow distribution toward more damaging cycles. Setting 1 uses the same low smoothing weight but a smaller tracking weight, producing a less severe stress spectrum. Settings 5 and 6, which use ${w}_{\Delta u}=100$, suppress rapid torque variation and consequently reduce the contribution of high-damage stress cycles. Setting 4 also uses the large smoothing penalty, but its poor speed transient generates a larger number of reversals and therefore does not achieve the same fatigue reduction as settings 5 and 6. The accumulated fatigue damage is determined through the combined application of Miner’s linear damage criterion and the S–N (Wöhler) fatigue behavior of the material. The fatigue results used in this assessment corresponds to the documented S–N characteristics of 18Ni700 maraging steel (Grade 250), specifically for the condition of finish-machined and subsequently maraged, as reported by the Nickel Institute^37^. Incorporating these material- specific fatigue properties ensure that the calculated damage values reflect not only the applied loading cycles but also the intrinsic endurance behavior of the steel, thereby providing a realistic estimate of cumulative fatigue deterioration under the various control settings.

**Fig. 15:**
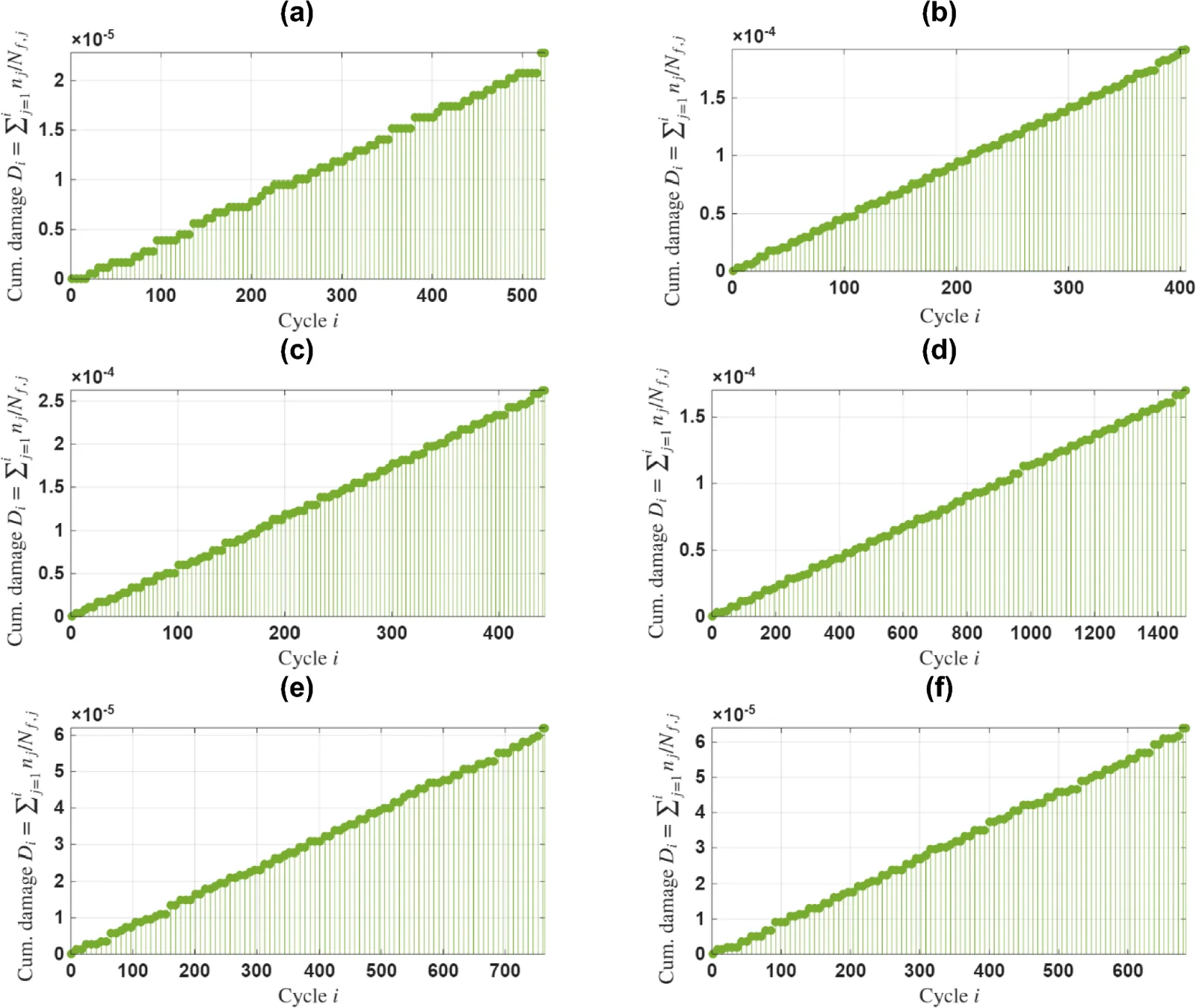
Cumulative damage from palmgren–miner rule for control (**a**) Setting 1 (**b**) Setting 2 (**c**) Setting 3 (**d**) Setting 4 (**e**) Setting 5 (**f**) Setting 6.

The cumulative-damage curves in Fig. 15 show an approximately monotonic increase with the number of counted cycles, as expected from the Palmgren–Miner linear-damage rule. The slope differs substantially among the control settings because each controller generates a different combination of stress ranges and mean stresses. Table 8 summarizes the record damage and corresponding fatigue-equivalent lives. The baseline controller gives a cumulative record damage of $1.83\times {10}^{-4}$, corresponding to a fatigue-equivalent life of *69.27* years under the assumed repeated-profile duty cycle. Setting 1 produces the lowest cumulative damage, $2.284\times {10}^{-5}$, which is approximately $87.5\mathrm{\%}$ below the baseline. Its corresponding fatigue-equivalent life is *555.43* years, approximately eight times the baseline value. Settings 2 and 3 produce damages of $1.922\times {10}^{-4}$ and $2.628\times {10}^{-4}$, resulting in lives of *65.99* and *48.27* years, respectively. Setting 3 therefore increases damage by approximately $43.6\mathrm{\%}$ relative to the baseline and gives the shortest fatigue-equivalent life among all cases. Setting 4 produces cumulative damage of $1.703\times {10}^{-4}$ and fatigue-equivalent life of *74.49* years. Although this is a modest improvement over the baseline, it is accompanied by the largest speed overshoot and settling time. Settings 5 and 6 produce substantially lower damage values of $6.20\times {10}^{-5}$ and $6.40\times {10}^{-5}$, corresponding to fatigue-equivalent lives of *204.58* and *198.11* years, respectively. Relative to the baseline, these settings reduce cumulative damage by approximately $66.1\mathrm{\%}$ and $65.0\mathrm{\%}$.

The fatigue-equivalent lives were obtained by repeating the *4.63*-day simulated stress record *78.83* times per year and assuming failure when the cumulative Miner damage reaches unity. These values should therefore be interpreted as comparative indicators of fatigue damage under an identical repeated operating profile. They do not represent the absolute service life of the complete FESS, because other failure mechanisms and uncertainties in geometry, material properties, manufacturing condition, and duty cycle are not included. The present investigation is based entirely on numerical simulation; therefore, the quantitative findings should be interpreted within the assumptions and limitations of the adopted AMB, PMSM, FESS, and fatigue models. As a partial benchmark for the fatigue-calculation procedure, the fatigue-equivalent lives reported in Table 8 were compared with the finite-element-based results of Nuñez et al.^23^ for a steel flywheel rotor subjected to grid-representative variable loading. Their study reported approximately 35,873 allowable operating cycles, corresponding to about 98 years at 6000 rpm, while failure was predicted when the maximum operating speed reached 10,000 rpm. In the present study, the baseline controller produced a fatigue-equivalent life of 69.27 years, while settings 2, 3, and 4 resulted in 65.99, 48.27, and 74.49 years, respectively. These values are within the same order of magnitude as the finite-element-based lifetime reported in^23^, providing indirect support for the plausibility of the adopted rainflow-counting, S–N, and Palmgren–Miner damage-calculation procedure. The substantially longer fatigue-equivalent lives obtained for settings 1, 5, and 6—555.43, 204.58, and 198.11 years, respectively—are associated with their lower controller-generated cumulative damage under the repeated-profile assumption. In particular, setting 1 reduced the cumulative damage to $2.284\times {10}^{-5}$, compared with $1.83\times {10}^{-4}$for the baseline controller. This comparison should not, however, be interpreted as direct validation of the absolute lifetime values. The present study and Ref^23^. differ in rotor geometry, material properties, operating-speed range, loading sequence, duty cycle, stress-analysis method, and cycle-to-year conversion assumptions. Accordingly, the comparison provides only an order-of-magnitude benchmark for the fatigue-damage calculation and does not validate the complete coupled AMB–PMSM–FESS dynamic response.

The combined dynamic and fatigue results confirm that controller tuning must be treated as a multi-objective problem. Increasing tracking weight while retaining a low input-smoothing penalty, as in settings 2 and 3, provides short settling times but increases AMB power consumption, magnetic loading, and cumulative fatigue damage. Applying a large smoothing penalty, as in settings 5 and 6, reduces fatigue damage and RMS torque, but generally increases the settling time. Setting 4 shows that excessive smoothing combined with a low tracking weight can produce an unacceptable speed overshoot despite a low RMS torque. Setting 1 provides the most favorable overall compromise for the investigated operating profile. It combines the lowest speed overshoot, lowest AMB power consumption, reduced bearing-force and displacement peaks, and the lowest cumulative fatigue damage. Settings 5 and 6 provide useful alternatives when stronger suppression of torque-rate variation is prioritized, but their longer transient responses and, in the case of setting 5, increased RMS angular activity should be considered. The results therefore demonstrate that fatigue-equivalent life should be evaluated together with tracking accuracy, settling time, rotor motion, actuator loading, and energy consumption when selecting MPC weights for an AMB-supported FESS.

## Conclusions

This study developed an integrated control-to-fatigue assessment framework for a flywheel energy storage system supported by active magnetic bearings. The framework links the PMSM torque command, flywheel-speed response, speed-dependent AMB dynamics, nominal centrifugal-stress history, rainflow cycle counting, and Palmgren–Miner cumulative damage. Six MPC weighting combinations were investigated and compared with a baseline controller to quantify the trade-off among speed tracking, torque smoothness, AMB actuation demand, rotor motion, power consumption, and fatigue-equivalent life. The results show that the controller weights have a substantial influence on both transient performance and long-term fatigue indicators. Setting 1 provided the most balanced overall response for the operating profile investigated. It reduced the speed overshoot from $10.66\mathrm{\%}$ for the baseline controller to $3.23\mathrm{\%}$, while maintaining a settling time of *90.06* s compared with *82.94* s for the baseline. Its RMS torque was $34.468 \mathrm{N}.\mathrm{m}$, representing a reduction of approximately $23.5\mathrm{\%}$ relative to the baseline value of $45.047 \mathrm{N}.\mathrm{m}$. Setting 1 also reduced the peak resultant magnetic force from $2.4794\times {10}^{4}$ to $2.0460\times {10}^{4}$ N, the peak resultant rotor displacement from $2.186\times {10}^{-3}$ to $1.884\times {10}^{-3}$ m, and the peak resultant AMB current from *70.710* to *65.054* A. In addition, the mean AMB power consumption decreased from *3718.7* to *1791.1* W, corresponding to a reduction of approximately $51.8\mathrm{\%}$. The quantitative comparison also shows that minimizing one performance index does not necessarily produce the best overall controller. Setting 4 gave the lowest RMS torque, $30.419 \mathrm{N}.\mathrm{m}$, but exhibited the largest speed overshoot, $22.12\mathrm{\%}$, and the longest settling time, *182.91* s. Setting 5 produced the lowest peak resultant magnetic force, $1.9915\times {10}^{4}$ N, and the lowest peak resultant rotor displacement, $1.880\times {10}^{-3}$ m; however, it also produced the largest RMS resultant rotor tilt, $2.990\times {10}^{-4}$ rad. Setting 3 imposed the greatest AMB energy demand, with a mean power consumption of *4741.5* W, and generated the highest cumulative fatigue damage among the investigated settings. The fatigue analysis further confirmed the importance of considering the complete stress-cycle distribution rather than relying only on overshoot or cycle count. Setting 1 produced the lowest cumulative damage, $2.284\times {10}^{-5}$, which is approximately $87.5\mathrm{\%}$ lower than the baseline value of $1.83\times {10}^{-4}$. Under the adopted repeated-profile assumption, its fatigue-equivalent life was *555.43* years, compared with *69.27* years for the baseline. Settings 5 and 6 also reduced cumulative damage to $6.20\times {10}^{-5}$ and $6.40\times {10}^{-5}$, corresponding to fatigue-equivalent lives of *204.58* and *198.11* years, respectively. In contrast, setting 3 produced the highest cumulative damage, $2.628\times {10}^{-4}$, and the shortest fatigue-equivalent life, *48.27* years. These results demonstrate that aggressive tracking combined with a low input-smoothing penalty can increase damaging stress amplitudes, whereas appropriate torque smoothing can substantially reduce cumulative fatigue damage.

Overall, setting 1 offered the most favorable compromise among tracking performance, AMB loading, rotor displacement, electrical consumption, and fatigue damage for the examined duty profile. Nevertheless, the reported lifetime values are comparative fatigue-equivalent indicators obtained by repeating the *4.63*-day simulated stress record *78.83* times per year and assuming failure at a Palmgren–Miner damage index of unity. They should not be interpreted as absolute service-life predictions for a complete FESS because the analytical solid-disk stress model does not include local geometric stress concentrations, shaft–hub effects, residual stresses, manufacturing variability, multiaxial loading, or other component failure mechanisms. Future work should therefore include finite-element stress validation, experimental or hardware-in-the-loop verification, formal stability and recursive-feasibility analysis of the speed-dependent AMB controller, and direct incorporation of fatigue-related quantities into the MPC objective function or constraints.

## Data Availability

The datasets used and/or analyzed during the current study are available from the corresponding author on reasonable request.
